# Supplementary material for: Randomised controlled trial of a Calcium Channel or Angiotensin Converting Enzyme Inhibitor/Angiotensin Receptor Blocker Regime to Reduce Blood Pressure Variability following Ischaemic Stroke (CAARBS): a protocol for a feasibility study
Source: BMJ Open. 2019 Feb 9;9(2):e025301. doi: 10.1136/bmjopen-2018-025301 (PMC6398677; doi:10.1136/bmjopen-2018-025301)
Supplement: Author's manuscript [file bmjopen-2018-025301.draft_revisions.pdf]

BMJ Open is committed to open peer review. As part of this commitment we make the peer review history of every article we publish publicly available.

When an article is published we post the peer reviewers' comments and the authors' responses online. We also post the versions of the paper that were used during peer review. These are the versions that the peer review comments apply to.

The versions of the paper that follow are the versions that were submitted during the peer review process. They are not the versions of record or the final published versions. They should not be cited or distributed as the published version of this manuscript.

BMJ Open is an open access journal and the full, final, typeset and author-corrected version of record of the manuscript is available on our site with no access controls, subscription charges or pay-per-view fees (http://bmjopen.bmj.com).

If you have any questions on BMJ Open's open peer review process please email editorial.bmjopen@bmj.com

BMJ Open

# A Randomised Controlled Trial of a Calcium Channel or Angiotensin Converting Enzyme Inhibitor/Angiotensin Receptor Blocker Regime to Reduce Blood Pressure Variability following Ischaemic Stroke (CAARBS) – a protocol for a feasibility study


| | |
|---|---|
| Journal: | *BMJ Open* |
| Manuscript ID | bmjopen-2018-025301 |
| Article Type: | Protocol |
| Date Submitted by the Author: | 09-Jul-2018 |
| Complete List of Authors: | Robinson, Tom; University of Leicester College of Medicine Biological Sciences and Psychology, Cardiovascular Sciences<br>Davison, William; University of East Anglia Faculty of Medicine and Health Sciences,<br>Rothwell, Peter; Stroke Prevention Research Unit, Nuffield Department of Clinical Neurosciences<br>Potter, John; University of East Anglia, Norwich Medical School; Norfolk and Norwich University Hospital, Stroke Research Group |
| Keywords: | blood pressure, blood pressure variability, Stroke < NEUROLOGY, cerebrovascular disease |
| | |


SCHOLARONE™
Manuscripts

# A Randomised Controlled Trial of a Calcium Channel or Angiotensin Converting Enzyme Inhibitor/Angiotensin Receptor Blocker Regime to Reduce Blood Pressure Variability following Ischaemic Stroke (CAARBS) – a protocol for a feasibility study

**Correspondence to:**

Dr William Davison

Clinical Research Fellow in Stroke Medicine

Bob Champion Research and Education Building,

James Watson Rd,

Norwich Research Park,

University of East Anglia,

Norwich,

NR4 7UQ

Tel: +44 (0) 1603 591245

Mail to: w.davison@uea.ac.uk

Keywords: blood pressure, blood pressure variability, stroke, cerebrovascular disease

Word count (excluding title page, refs, tables, figures): 3831 words

Authors:

Professor Thompson G Robinson, Professor of Stroke Medicine, University of Leicester, Leicester, UK

Dr William J Davison, Clinical Research Fellow in Stroke Medicine, University of East Anglia, Norwich, UK

Professor Peter M Rothwell, Action Research Professor of Clinical Neurology, University of Oxford, Oxford, UK

Professor John F Potter, Professor of Ageing & Stroke Medicine, University of East Anglia, Norwich, UK

Author Contributions:

For peer review only

T Robinson, J Potter and P Rothwell obtained funding for the project and designed the study as part of a programme of work on blood pressure variability and stroke. The protocol was written by T Robinson and W Davison and reviewed by the other contributing authors. This manuscript has been prepared by W Davison and adapted from protocol version 1.1 dated 11/9/17. The final manuscript has been reviewed by all authors and approved for submission/publication.

For peer review only

ABSTRACT

Introduction: Raised blood pressure (BP) is common post-stroke and is associated with a poor prognosis, yet trials of BP lowering in the immediate post-stroke period have not demonstrated a benefit. One possible explanation for this may be that BP variability (BPV) rather than absolute levels predicts outcome, as BPV is increased post-stroke and is associated with poor outcomes. Furthermore, there is evidence of distinct antihypertensive class effects on BPV despite similar BP lowering effects. However, whether BPV in the immediate post-stroke period is a therapeutic target has not been prospectively investigated.

The objectives of this trial are to assess the feasibility and safety of recruiting patients following an acute ischaemic stroke or transient ischaemic attack (TIA) to an interventional randomised controlled trial comparing the effects of two different antihypertensive drug classes on BPV. Secondary exploratory objectives are to assess if different therapeutic strategies have diverse effects on levels of BPV and if this has an impact on outcomes.

Methods: 150 adult patients with first-ever ischaemic stroke or TIA who require antihypertensive therapy for secondary prevention will be recruited within 72 hours of the event from stroke services across three sites. After baseline assessments they will be randomly assigned to treatment with a calcium channel blocker or angiotensin converting enzyme inhibitor/angiotensin receptor blocker based regimen and followed-up for a period of three months.

Ethics and dissemination: Ethical and regulatory approvals have been granted. Dissemination is planned via publication in peer-reviewed medical journals and presentation at relevant conferences.

Registration details: International standard randomised controlled trial number (ISRCTN) 10853487.

For peer review only

**Strengths and limitations of this study:**

- To our knowledge this is the first prospective randomised trial designed to assess the treatment of BPV following acute ischaemic stroke/TIA.
- The protocol incorporates multiple BP measurement methods.
- The chosen therapeutic interventions are in line with standard clinical practice for secondary stroke prevention.

- The trial is open-label which could bias the analysis of treatment effects on BPV and any impact on stroke outcomes, but these are secondary exploratory outcomes in this feasibility trial.

INTRODUCTION

**Background**

Raised BP is common after acute stroke with at least 75% of patients having a systolic BP (SBP) >130mmHg at hospital admission [1, 2]; SBP <130mmHg being the guideline target for secondary prevention following stroke [3]. Increased post-stroke BP is associated with poor prognosis [4, 5] and may result from raised intracranial pressure [6], increased sympathetic nervous system activity [7], abnormal baroreceptor sensitivity (BRS) [8], haematoma expansion [9], cerebral oedema [10], and a white-coat response [11]. A spontaneous BP decrease usually occurs 4 to 10 days post-ictus [12], but substantial BP reductions can be associated with cerebral hypoperfusion as a consequence of post-stroke dysautoregulation [13]. We have previously reported that both increased 24-hour and beat-to-beat BP levels following acute stroke are associated with a poor prognosis [14-16]. Subsequently, data from the International Stroke Trial has suggested a U-shaped relation between baseline SBP (within 48 hours of stroke) and short- (14-day mortality) and long-term (6-month death and dependency) outcomes; the lowest risk of death and dependency being at SBP of 150mmHg [17]. However, there is conflicting evidence regarding acute stroke hypertension treatment. Data from randomised controlled trials (RCTs) suggest that BP can be safely reduced after the acute stroke period, however, there seems to be no indication that doing so is beneficial. [18-23]. Indeed, the SCAST trial reported that it may actually be harmful, with a non-significant increased risk of poor 6-month functional outcome [23]. Therefore, Cochrane meta-analysis and guidelines state that optimal BP management in the context of initial stroke management remains uncertain [3, 24-26].

An alternative explanation for the lack of evidence that lowering elevated BP levels in acute stroke is beneficial may relate to the additional effects of BPV [27]. Current hypertension guidelines predominantly focus on mean, usually casual, BP measurements, dismissing BPV as random and merely an obstacle to the reliable estimation of usual BP. However, on ambulatory or home BP monitoring, which are recommended for the diagnosis and management of hypertension [28], mean BP is found to vary substantially [29], with the extent of this variation associated with visit-to-visit variability in clinic BP [30]. Indeed, there are many examples to support the potential importance of

BPV for vascular risk [30]. Firstly, the predictive value of estimated usual SBP and stroke risk falls with age [31], yet stroke incidence rises with age and the relative benefit of antihypertensive therapy is maintained in the elderly [32]. Secondly, an increased early-morning surge in BP is predictive of stroke, but is poorly associated with mean BP [33]. Thirdly, other causes of transient hypertension are recognised triggers of vascular events, including sympathetic overactivity and orthostatic hypertension [34]. Fourthly, in the majority of studies, there is no threshold of baseline SBP below which vascular risk stops falling (though evidence for BP below 115/75mmHg is very limited) [31], with antihypertensive therapy reducing risk even at 'normal' baseline SBP [35]. Fifthly, 'white-coat' hypertension, a common example of situational BPV, is associated with long-term target organ damage independent of mean BP [36]. Sixthly, though hypertension is a recognised risk factor for vascular dementia, there is limited evidence of reduced dementia risk in trials of antihypertensive therapy. However, a trial of calcium channel blockers (CCB), which have the most consistent effect on reducing BPV [37, 38], has shown a substantial reduction in the incidence of dementia [39]. Finally, specific group differences in stroke risk are not accounted for by mean BP alone, for example in black individuals [40].

**Rationale for the study**

In a retrospective analysis of RCTs in a TIA population, visit-to-visit intra-individual BPV was a risk factor for stroke independent of the mean 'absolute' BP level, and perhaps of greater significance [30]. Additionally, within-visit systolic BPV, based on casual BP measurements, was correlated with visit-to-visit systolic BPV, but was a weak predictor of future vascular events [30]. Importantly, in a separate analysis it was demonstrated that BPV is reproducible and independent of confounding factors [41]. Increased BPV may also be an important predictor of short-term outcome following acute stroke. Robinson and colleagues have shown that beat-to-beat systolic BPV was greater in acute stroke compared to controls [42], and that high mean arterial and diastolic beat-to-beat BPV was associated with a worse prognosis [15]. Furthermore, in a post hoc analysis of the Tinzaparin in Acute Ischaemic Stroke Trial (TAIST), high systolic BPV from 3-6 casual BP readings, taken within 48 hours of symptom onset, was associated with an increase in death or early neurological deterioration at day 10 [43]. Conversely, a retrospective analysis of nearly 1,000 patients in the Continue or Stop Post-Stroke Antihypertensives Collaborative Study (COSSACS) and Controlling Hypertension and Hypotension Immediately Post Stroke (CHHIPS) trial did not demonstrate a significant association between systolic BPV based on two sets of three casual BP readings within 48 hours of stroke onset and two-week death and dependency [44]. Overall, a recent meta-analysis reported that increased systolic BPV, measured early from stroke onset, was associated with poor

long-term functional outcome [45]. Furthermore, increased BPV may also relate to post-stroke cognitive outcomes with evidence suggesting an association with signs of cerebrovascular small vessel disease on neuroimaging [46], and deterioration in cognitive test scores [47, 48].

Clearly there is further scope to explore the relationship between BPV and outcome following acute stroke, in particular whether it has implications for therapeutic management in the immediate post-stroke period. Rothwell's group have explored the differential effects of BP-lowering therapies on BPV in a hypertensive population [37, 38]. Though clinical benefits with reduction in risk of stroke and coronary events were seen for all classes of antihypertensive agent, class-specific effects existed; CCBs reduce stroke risk to a greater extent than expected from mean SBP reduction alone, and beta-blockers (BB) to a smaller extent. A detailed analysis of the Anglo-Scandinavian Cardiac Outcomes Trial – Blood Pressure Lowering Arm (ASCOT-BPLA), comparing an amlodipine versus atenolol-based regime, and the Medical Research Council (MRC) trial, comparing an atenolol versus diuretic-based regime, reported opposite effects of CCB and BB on systolic BPV. In addition, this differential effect accounted for the disparity in observed effects on stroke risk and observed effects on mean SBP [38]. This was confirmed in a systematic review and meta-analysis of 389 RCTs; Webb and colleagues reporting that systolic BPV was significantly reduced by CCB and non-loop diuretic drugs, but increased by angiotensin converting enzyme inhibitors (ACEI), angiotensin receptor blockers (ARB) and BB [37]. Again, the effects on systolic BPV were correlated with effects on stroke risk independent of differences in mean SBP [37]. The potential differential effect of antihypertensive drug classes on BPV is possibly important after acute stroke, where normal cardiovascular autonomic and cerebrovascular autoregulatory pathways are impaired. BRS is important in the short-term regulation of the cardiovascular system, including BP, and is known to be impaired following acute ischaemic stroke [8], and associated with poor short and long-term prognosis [49]. In addition, it is well established that cerebral autoregulation (CA) is impaired, particularly following moderate to severe stroke [13]. As a consequence, cerebral perfusion is pressure-dependent, and therefore hypertensive episodes related to increased BPV may contribute to reperfusion injuries, for example post-ischaemic oedema and/or intracerebral haemorrhage. Conversely, hypotensive episodes associated with increased BPV in the presence of impaired CA may lead to secondary ischaemia, particularly in the absence of a good collateral circulation.

In conclusion, increased BPV is associated with a greater vascular risk, independent of mean BP and may predict poor outcomes after stroke. Furthermore, commonly used antihypertensive agents have different class effects on BPV which may in part explain the overall differential effects on stroke risk

For peer review only

for similar absolute reductions in mean BP in a hypertensive population. Trials to investigate the potential therapeutic targeting of BPV and any potential benefit of doing so in acute stroke would be useful to address gaps in the current knowledge base.

**Study objectives**

The primary objective of this study is to determine feasibility of recruiting patients with acute stroke and TIA into an interventional randomised trial comparing the effect of different antihypertensive medication regimens on BPV.

Secondary feasibility objectives are:

- to determine the viability of measuring changes in BPV from baseline to 21 (±7) days and 90 (±14) days by treatment arm;
- to assess compliance rates with BPV measurement methods;
- to assess compliance rates with the investigational treatments;
- to identify serious adverse events (SAEs) associated with the interventions, including recurrent stroke/TIA, other cardiovascular events, death, and hospital readmission up to three months.

In addition to the feasibility objectives, exploratory outcomes that may be used in a future definitive RCT will be investigated. The proposed primary exploratory outcome will be modified Rankin score (mRS) at day 90.

Exploratory secondary outcomes are:

- mRS at day 21;
- National Institutes of Health Stroke Scale (NIHSS) at day 21;
- mean BP at day 21 and day 90;
- BPV at day 21 day and 90;
- Montreal cognitive assessment (MoCA) score at day 90.

## METHODS AND ANALYSIS

**Study overview**

For peer review only

This study is a randomised, multi-centre, open-label parallel group study to determine the feasibility of conducting such a trial in an NHS setting to investigate class effects of antihypertensive medications on BPV in patients with acute ischaemic stroke or TIA. The aim is to evaluate barriers to recruitment, identify potential safety issues, and demonstrate that it is possible to detect differences in BPV over the proposed study duration. We also hope to investigate the potential therapeutic benefit of targeting BPV after acute ischaemic stroke/TIA in terms of functional outcome in order to help estimate the necessary sample size for a future definitive trial. A summary of the study design is provided in **Figure 1.** Recruitment commenced in January 2018 and is ongoing. The trial was prospectively registered: International standard randomised controlled trial number (ISRCTN) 10853487.

**Trial Participants**

All adult patients with clinically definite first-ever ischaemic stroke or TIA within 72 hours of onset will be considered for the trial.

**Inclusion Criteria**

- Age >18 years;
- First-ever clinically definite TIA and ischaemic stroke patients (NIHSS <10);
- Within 72 hours of symptom onset;
- Casual BP >130/80mmHg on repeat measurements;
- Ability to comply with randomly assigned BP-lowering regime and BP measurements;
- Able to understand written and verbal English;
- Able to give informed consent;
- Willing to allow his or her General Practitioner and consultant, if appropriate, to be notified of participation in the study.

**Exclusion Criteria**

The participant may not enter the trial if any of the following apply:

- Known definite contra-indication to BP-lowering regime or therapeutic agents;
- Swallowing difficulties which would preclude the taking of oral medication;
- Definite indication for BB, CCB, ACEI or ARB therapy;
- Significant pre-stroke dependency (mRS >3);
- Co-existing life-threatening condition with life expectancy <3 months;

For peer review only

For peer review only - http://bmjopen.bmj.com/site/about/guidelines.xhtml

- Previous participation in this trial or current participation in another investigational drug trial;
- Atrial fibrillation;
- Female participants who are pregnant, lactating or planning pregnancy during the course of the study;
- Unable to understand written and verbal English;
- Cannot give informed consent.

**Identification of participants**

First-ever TIA and minor ischaemic stroke patients referred to and assessed by the in- and/ or out-patients stroke services at three centres within 72 hours of symptom onset will be identified by the treating clinician and/ or the research team. If the patient provides verbal consent to be considered for the study then their medical records will then be assessed against the study inclusion and exclusion criteria. Patients known to be hypertensive and on treatment prior to their cerebrovascular event should have their antihypertensive medications suspended at admission, in keeping with standard practice at the recruiting centres, unless there is a specific indication for them to continue. Where treatment is suspended and the patient is willing to be considered for the trial then they are potentially eligible for inclusion provided other inclusion/exclusion criteria are not violated. Once a potential participant has been confirmed to be eligible then research staff will approach the individual to discuss the study in more detail, provide a Participant Information Sheet, and seek written informed consent.

**Obtaining informed consent**

The participant must personally sign and date the latest approved version of the informed consent form, countersigned by a delegated member of the research team, before any study specific procedures are performed. Written and verbal versions of the participant information sheet and Informed consent form will be presented to the participants detailing no less than: the exact nature of the study; the implications and constraints of the protocol; the known side effects and any risks involved in taking part. It will be clearly stated that the participant is free to withdraw from the study at any time for any reason without prejudice to future care, and with no obligation to give the reason for withdrawal. The person who obtained the consent must be suitably qualified and experienced, and have been authorised to do so by the Chief/Principal Investigator as detailed on the Delegation of Authority and Signature log for the study. The original signed form will be retained at the study site within the Investigator Site File. A copy of the signed Informed Consent will be given to participants

For peer review only

and a copy retained in their medical notes.

## Randomisation

After the baseline assessments eligible patients will be randomised using a computer generated protocol, in blocks of four, to a dihydropyridine CCB or ACEI/ARB-based regime. The study treatment will be dispensed at the baseline visit, but treatment will not be commenced within 48 hours of the qualifying event in keeping with current recommended practice. The actual therapeutic agent used will be at the discretion of the treating clinician, but dictated by the class of therapy that the participant is assigned to. Prescription of the medication will be done by the treating clinician and the initial supply will be dispensed by the treating hospital or community pharmacy in accordance with the hospital's policy for providing discharge or out-patient medication. Further supplies will be provided by the participant's GP. Unblinding will not be necessary as there is an open-label study design.

## Interventions to be measured

### *Routine clinical data*

The following routine clinical information and investigation results will be obtained from the medical notes and by participant interview:

- Demographics (including age, sex, ethnicity, height and weight, smoking and alcohol habits);
- Past medical history and family history of cardiovascular disease;
- Concomitant medications;
- NIHSS;
- mRS (including pre-morbid mRS);
- Oxford Community Stroke Project and TOAST classification;
- Laboratory tests (including full blood count, clotting, urea, electrolytes, creatinine, estimated glomerular filtration rate, total cholesterol, and random glucose);
- 12-lead ECG (± 24-hour ECG if performed);
- Imaging investigations (including neuroimaging (CT or MRI), carotid ultrasound, and cardiac echocardiography where applicable).

### *Blood pressure measurements*

Baseline casual BP will be calculated as a mean of two sets of three supine brachial BP readings taken 10 minutes apart, using a UA767 BP monitor (referred to as enhanced casual BP).

Three consecutive periods of 10-min beat-to-beat non-invasive BP monitoring in the supine position using the middle finger of the non-hemiparetic hand will be recorded with a Finometer device. The

For peer review only

servo adjust mechanism of the Finometer will be switched off during the recording period, but applied at 10 minute intervals during the monitoring period.

Daytime ABPM will be performed using a SpaceLabs 90207 monitor, programmed to measure BP at 20-minute intervals. Daytime is defined as between 0700-2200 hours.

***Cognitive testing***

A battery of cognitive tests will be performed. This will include the MoCA screening test which is established for use after cerebrovascular events, augmented with the Albert's line test for inattention, the Motor Neuron Disease Behavioural Instrument (MiND-B) for frontal cognitive symptoms, and the Geriatric Depression Scale (GDS) to exclude significant concurrent anxiety/depression.

**Follow-up Assessments**

These will be undertaken at day 21 (±7 days) and day 90 (±14 days) in the trial centre or where the patient is resident at the time (including the hospital ward, rehabilitation facility, or their own home). Interventions that will repeated at these follow-up visits are summarised in **Table 1**. Additional follow-up interventions to assess the trial feasibility and safety will include assessment of treatment compliance using a self-reported questionnaire and tablet count (with compliance defined as ≥80%), and assessment of any side-effects and SAEs. Patients randomised to the ACEI/ARB arm will have repeat renal function blood tests at the first follow-up visit in line with standard practice to ensure their safety. In those patients failing to reach casual supine/sitting BP target of <130/ 80mmHg, the medical assessor at the follow-up visit will advise about altering BP-lowering treatment and this will be communicated to the participant's GP. The first-line change will be to increase the study regime medication (i.e. CCB or ACEI/ARB) to twice the starting dose. If the patient is on the maximum dose of the study regime medication already, then the second-line change will be to add a thiazide-like diuretic. If a third-line change is required then Spironolactone or an alpha-blocker will be added to the combination of study medication and thiazide-like diuretic. After the second follow-up visit ongoing management of the patient's BP will be taken over by the GP.

**Table 1: Summary of trial procedures**

| Procedures | **Visits** | | | |
|---|---|---|---|---|
| | **Screening** | **Baseline** | **21 (±7) days** | **90 (±14) days** |
| Informed consent | | **X** | | |
| Demographics | | **X** | | |

| | | | | |
|---|---|---|---|---|
| Medical history | | X | | |
| Concomitant medications | | X | X | X |
| ECG | | X | | |
| Clinical investigation results (bloods tests, CT/MRI scan results) | | X | | |
| Eligibility assessment | X | | | |
| Randomisation | | X | | |
| Dispensing of study drugs | | X | | |
| Treatment compliance | | | X | X |
| Blood test for renal function in ACEI/ARB group | | | X | |
| NIHSS | | X | X | X |
| mRS | | X[1] | X | X |
| MoCA | | X | | X |
| Albert's line test | | X | | X |
| MiND-B | | X | | X |
| GDS | | X | | X |
| Enhanced casual BP | | X | X | X |
| Beat-to-beat BP measurements | | X | X | X |
| Daytime ABPM | | X | | X |
| SAEs | | | X | X[2] |

[1] Including Premorbid mRS

[2] SAEs at Day 90 followed-up until resolution

For peer review only

**Outcome measurements**

1. Primary feasibility outcome measure:

Recruitment and retention rates at three months from the screening and management logs, and reasons for ineligibility or non-inclusion of those screened but not recruited.

2. Secondary feasibility outcome measures:

(a) changes in BPV from baseline to 21 (+7) days and 90 (+14) days by treatment arm;

(b) proportions of participants achieving ≥80% treatment compliance by treatment arm;

(c) treatment discontinuation rates;

(d) completion and failure rates of BPV measurements at 21 (±7) days and 90 (±14) days;

(e) serious adverse event rates by treatment arm.

3. Exploratory outcome measures:

(a) mRS at 90 (±14) days by treatment arm;

(b) mRS at 21 (±7) days by treatment arm;

(c) NIHSS at 21 (±7) days by treatment arm;

(d) differences in mean BP at 21 (±7) days and 90 (±14) days by treatment arm;

(e) differences in BPV at 21 (±7) days and 90 (±14) days by treatment arm;

(f) differences in MoCA score at 90 (±14) days by treatment arm.

**Sample size calculation**

A feasibility study of 150 patients (64 patients per group with a 15% drop-out rate) will have an 80% power at the 5% significance level of detecting an 8mmHg difference in systolic BPV between the CCB and ACEI/ARB-based regimes, assuming a mean systolic BPV SD of 14.97mmHg in the CCB arm and 16.95mmHg in the ACEI/ARB arm [37].

**Data analysis plan**

The primary objective is assessment of feasibility. This will focus on recruitment and retention rates, compliance, change in BPV, and safety of the intervention. Exploratory analysis of the effect of the proposed intervention on BPV and stroke outcome will be done as a secondary objective.

***Recruitment and Retention***

The total numbers of patients screened, the proportion recruited, and the proportion completing follow-up will be determined. Reasons for ineligibility, non-inclusion, and withdrawal will be analysed using descriptive statistical methods.

***Assessment of the intervention***

Compliance with the intervention will be assessed by the proportion of participants who achieve ≥80% adherence to the trial medication and the proportion of participants who have all BP measurements recorded successfully.

The feasibility of detecting changes in BPV will also be assessed. Within-individual systolic, diastolic and mean BPV will be expressed as the standard deviation, coefficient of variation, average real variability, and variation independent of the mean calculated from all BP measurements: enhanced

For peer review only

casual, beat-to-beat measurements (each 10 minute recording and the total 30 minute recording), and daytime ABPM [41]. Changes in within-individual BPV from baseline to the follow-up time points will be analysed using a general linear model. The size of the mean difference will be estimated for each approach and compared to select the most appropriate measure for a future study.

***Safety***

Rates of serious adverse events, including recurrent stroke/TIA, other cardiovascular events, death, and hospitalization will be recorded up to 3 months. A descriptive comparison will be undertaken to compare the rates, but no formal hypothesis testing will be undertaken.

***Exploratory Analyses***

Mean BP will be calculated from enhanced casual measurements. Change in mean BP from baseline to follow-up by treatment arm will be compared using an independent samples T test.

An assessment of treatment effect on BPV will be undertaken stratified according to treatment arm. A general linear model will be used with BPV as the dependent variable and treatment arm as the independent variable, adjusting for baseline BP and diagnosis (stroke vs. TIA). Each expression of BPV as described above will be analysed.

Exploratory assessment of treatment effect on stroke outcome will be undertaken by comparing between-group differences in mRS and MoCA score at follow-up using independent samples T tests or a non-parametric test if the assumptions of the t-test are violated.

ETHICS AND DISSEMINATION

This study was granted ethical approval in England (London - Central Research Ethics Committee, REC 17/LO/1427) and clinical trial authorisation from the Medicines and Healthcare products Regulatory Agency (EudraCT number 2017-002560-41). Subsequently the trial was approved by the Health Research Authority. Study oversight will be conducted through regular meetings of a Trial Steering Committee and a separate Safety Committee, both of which will include independent representatives. If it is felt that the risk to participants is significant or unacceptable the Safety Committee can recommend to early termination of the trial.

The proposed investigational medicinal products are antihypertensives that are already in routine use and so their safety profiles are known. The medications are expected to lower the BP of

For peer review only

participants. Therefore, in line with accepted stroke guidelines we will only recruit patients with uncontrolled BP (>130/ 80mmHg) who would otherwise require antihypertensive treatment for secondary stroke prevention. Medications that inhibit the renin-angiotensin system are known to potentially cause kidney dysfunction in patients with unrecognised renal artery stenosis. To ensure the safety of patients commenced on these medications a blood test for kidney function will be done at the 2 to 4-week follow-up which is in keeping with standard practice.

The trial will be conducted in accordance with the Declaration of Helsinki and the ICH Guidelines for Good Clinical Practice. All participants will provide written informed consent. Data will be collected and handled in line with sponsor standard operating procedures and NHS Trust policies. Electronic data will be anonymised and all data will be kept under secure conditions. Professor Robinson will act as data custodian.

Dissemination of the study results is planned via publication in peer-reviewed medical journals and presentation at relevant scientific conferences. Any reporting will adhere to the CONSORT statement extension for pilot and feasibility trials. We do not intend to employ professional writers.

Competing Interests: Professor Robinson and Professor Rothwell are both NIHR Senior Investigators.

This work was supported by a programme grant awarded jointly by The Stroke Association and The British Heart Foundation (Ref: TSA BHF 2012/01).

The study is sponsored by the University of Leicester. The sponsor has not had a role in the design or implementation of the study, nor the writing of this protocol.

Contact details for the sponsor:
Dr Michelle Muessel
Research Governance Manager
Research Governance Office
Research and Enterprise Division
University of Leicester
Leicester General Hospital
Gwendolen Rd
Leicester LE5 4PW

For peer review only

Tel: 0116 2584099/2584867

Mail to: uolsponsor@le.ac.uk

**References**


1. IST Collaborative Group, *The International Stroke Trial (IST): a randomised trial of aspirin, subcutaneous heparin, both, or neither among 19435 patients with acute ischaemic stroke. .* Lancet, 1997. **349**(9065): p. 1569-81.
2. CAST (Chinese Acute Stroke Trial) Collaborative Group, *CAST: randomised placebo-controlled trial of early aspirin use in 20,000 patients with acute ischaemic stroke.* Lancet, 1997. **349**(9066): p. 1641-9.
3. Intercollegiate Stroke Working Party. *National Clinical Guideline for Stroke 5th ed.* 2016 [cited 2017 17th July 2017]; Available from: https://www.rcplondon.ac.uk/guidelines-policy/stroke-guidelines.
4. Willmot, M., J. Leonardi-Bee, and P.M. Bath, *High blood pressure in acute stroke and subsequent outcome: a systematic review.* Hypertension, 2004. **43**(1): p. 18-24.
5. Tikhonoff, V., H. Zhang, T. Richart, and J.A. Staessen, *Blood pressure as a prognostic factor after acute stroke.* Lancet Neurol, 2009. **8**(10): p. 938-48.
6. Fodstad, H., P.J. Kelly, and M. Buchfelder, *History of the cushing reflex.* Neurosurgery, 2006. **59**(5): p. 1132-7; discussion 1137.
7. Chamorro, A., S. Amaro, M. Vargas, V. Obach, A. Cervera, M. Gomez-Choco*, et al.*, *Catecholamines, infection, and death in acute ischemic stroke.* J Neurol Sci, 2007. **252**(1): p. 29-35.
8. Robinson, T.G., M. James, J. Youde, R. Panerai, and J. Potter, *Cardiac baroreceptor sensitivity is impaired after acute stroke.* Stroke, 1997. **28**(9): p. 1671-6.
9. Ohwaki, K., E. Yano, H. Nagashima, M. Hirata, T. Nakagomi, and A. Tamura, *Blood pressure management in acute intracerebral hemorrhage: relationship between elevated blood pressure and hematoma enlargement.* Stroke, 2004. **35**(6): p. 1364-7.
10. Qureshi, A.I., *Acute hypertensive response in patients with stroke: pathophysiology and management.* Circulation, 2008. **118**(2): p. 176-87.
11. Carlberg, B., K. Asplund, and E. Hagg, *High blood pressure in acute stroke--is it white coat hypertension?* J Intern Med, 1990. **228**(3): p. 291-2.
12. Britton, M., A. Carlsson, and U. de Faire, *Blood pressure course in patients with acute stroke and matched controls.* Stroke, 1986. **17**(5): p. 861-4.
13. Aries, M.J., J.W. Elting, J. De Keyser, B.P. Kremer, and P.C. Vroomen, *Cerebral autoregulation in stroke: a review of transcranial Doppler studies.* Stroke, 2010. **41**(11): p. 2697-704.
14. Robinson, T., A. Waddington, S. Ward-Close, N. Taub, and J. Potter, *The predictive role of 24-hour compared to casual blood pressure levels on outcome following acute stroke.* Cerebrovasc Dis, 1997. **7**: p. 264-272.
15. Dawson, S.L., B.N. Manktelow, T.G. Robinson, R.B. Panerai, and J.F. Potter, *Which parameters of beat-to-beat blood pressure and variability best predict early outcome after acute ischemic stroke?* Stroke, 2000. **31**(2): p. 463-468.
16. Webb, A.J.S., S. Mazzucco, L. Li, and P.M. Rothwell, *Prognostic Significance of Blood Pressure Variability on Beat-to-Beat Monitoring After Transient Ischemic Attack and Stroke.* Stroke, 2018. **49**(1): p. 62-67.
17. Leonardi-Bee, J., P.M. Bath, S.J. Phillips, P.A. Sandercock, and I.S.T.C. Group, *Blood pressure and clinical outcomes in the International Stroke Trial.* Stroke, 2002. **33**(5): p. 1315-20.


For peer review only

18. Schrader, J., S. Luders, A. Kulschewski, J. Berger, W. Zidek, J. Treib, *et al.*, *The ACCESS Study: evaluation of Acute Candesartan Cilexetil Therapy in Stroke Survivors.* Stroke, 2003. **34**(7): p. 1699-703.
19. Anderson, C.S., Y. Huang, J.G. Wang, H. Arima, B. Neal, B. Peng, *et al.*, *Intensive blood pressure reduction in acute cerebral haemorrhage trial (INTERACT): a randomised pilot trial.* Lancet Neurol, 2008. **7**(5): p. 391-9.
20. Potter, J.F., T.G. Robinson, G.A. Ford, A. Mistri, M. James, J. Chernova, *et al.*, *Controlling hypertension and hypotension immediately post-stroke (CHHIPS): a randomised, placebo-controlled, double-blind pilot trial.* Lancet Neurol, 2009. **8**(1): p. 48-56.
21. Bath, P.M., R.H. Martin, Y. Palesch, D. Cotton, S. Yusuf, R. Sacco, *et al.*, *Effect of telmisartan on functional outcome, recurrence, and blood pressure in patients with acute mild ischemic stroke: a PRoFESS subgroup analysis.* Stroke, 2009. **40**(11): p. 3541-6.
22. Robinson, T.G., J.F. Potter, G.A. Ford, C.J. Bulpitt, J. Chernova, C. Jagger, *et al.*, *Effects of antihypertensive treatment after acute stroke in the Continue or Stop Post-Stroke Antihypertensives Collaborative Study (COSSACS): a prospective, randomised, open, blinded-endpoint trial.* Lancet Neurol, 2010. **9**(8): p. 767-75.
23. Sandset, E.C., P.M. Bath, G. Boysen, D. Jatuzis, J. Korv, S. Luders, *et al.*, *The angiotensin-receptor blocker candesartan for treatment of acute stroke (SCAST): a randomised, placebo-controlled, double-blind trial.* Lancet, 2011. **377**(9767): p. 741-50.
24. Bath, P.M. and K. Krishnan, *Interventions for deliberately altering blood pressure in acute stroke.* Cochrane Database Syst Rev, 2014(10): p. CD000039.
25. National Institute for Health and Care Excellence. *Stroke and transient ischaemic attack in over 16s: diagnosis and initial management*. 2008 [cited 2017 17th July 2017]; Available from: https://www.nice.org.uk/guidance/CG68.
26. Kernan, W.N., B. Ovbiagele, H.R. Black, D.M. Bravata, M.I. Chimowitz, M.D. Ezekowitz, *et al.*, *Guidelines for the prevention of stroke in patients with stroke and transient ischemic attack: a guideline for healthcare professionals from the American Heart Association/American Stroke Association.* Stroke, 2014. **45**(7): p. 2160-236.
27. Rothwell, P.M., *Limitations of the usual blood-pressure hypothesis and importance of variability, instability, and episodic hypertension.* Lancet, 2010. **375**(9718): p. 938-48.
28. National Institute for Health and Care Excellence. *Hypertension in adults: diagnosis and management*. 2011 [cited 2017 17th July 2017]; Available from: www.nice.org.uk/guidance/cg127.
29. Wizner, B., D.G. Dechering, L. Thijs, N. Atkins, R. Fagard, E. O'Brien, *et al.*, *Short-term and long-term repeatability of the morning blood pressure in older patients with isolated systolic hypertension.* J Hypertens, 2008. **26**(7): p. 1328-35.
30. Rothwell, P.M., S.C. Howard, E. Dolan, E. O'Brien, J.E. Dobson, B. Dahlof, *et al.*, *Prognostic significance of visit-to-visit variability, maximum systolic blood pressure, and episodic hypertension.* Lancet, 2010. **375**(9718): p. 895-905.
31. Lewington, S., R. Clarke, N. Qizilbash, R. Peto, R. Collins, and C. Prospective Studies, *Age-specific relevance of usual blood pressure to vascular mortality: a meta-analysis of individual data for one million adults in 61 prospective studies.* Lancet, 2002. **360**(9349): p. 1903-13.
32. Beckett, N.S., R. Peters, A.E. Fletcher, J.A. Staessen, L. Liu, D. Dumitrascu, *et al.*, *Treatment of hypertension in patients 80 years of age or older.* N Engl J Med, 2008. **358**(18): p. 1887-98.
33. Kario, K., K. Shimada, and T.G. Pickering, *Clinical implication of morning blood pressure surge in hypertension.* J Cardiovasc Pharmacol, 2003. **42 Suppl 1**: p. S87-91.
34. Kario, K., *Orthostatic hypertension: a measure of blood pressure variation for predicting cardiovascular risk.* Circ J, 2009. **73**(6): p. 1002-7.
35. Progress Collaborative Group, *Randomised trial of a perindopril-based blood-pressure-lowering regimen among 6,105 individuals with previous stroke or transient ischaemic attack.* Lancet, 2001. **358**(9287): p. 1033-41.

For peer review only

36. Mancia, G., M. Bombelli, R. Facchetti, F. Madotto, F. Quarti-Trevano, H. Polo Friz*, et al.*, *Long-term risk of sustained hypertension in white-coat or masked hypertension.* Hypertension, 2009. **54**(2): p. 226-32.
37. Webb, A.J., U. Fischer, Z. Mehta, and P.M. Rothwell, *Effects of antihypertensive-drug class on interindividual variation in blood pressure and risk of stroke: a systematic review and meta-analysis.* Lancet, 2010. **375**(9718): p. 906-15.
38. Rothwell, P.M., S.C. Howard, E. Dolan, E. O'Brien, J.E. Dobson, B. Dahlof*, et al.*, *Effects of beta blockers and calcium-channel blockers on within-individual variability in blood pressure and risk of stroke.* Lancet Neurol, 2010. **9**(5): p. 469-80.
39. Forette, F., M.L. Seux, J.A. Staessen, L. Thijs, W.H. Birkenhager, M.R. Babarskiene*, et al.*, *Prevention of dementia in randomised double-blind placebo-controlled Systolic Hypertension in Europe (Syst-Eur) trial.* Lancet, 1998. **352**(9137): p. 1347-51.
40. Duprez, D.A., D.R. Jacobs, Jr., P.L. Lutsey, D. Herrington, D. Prime, P. Ouyang*, et al.*, *Race/ethnic and sex differences in large and small artery elasticity--results of the multi-ethnic study of atherosclerosis (MESA).* Ethn Dis, 2009. **19**(3): p. 243-50.
41. Howard, S.C. and P.M. Rothwell, *Reproducibility of measures of visit-to-visit variability in blood pressure after transient ischaemic attack or minor stroke.* Cerebrovasc Dis, 2009. **28**(4): p. 331-40.
42. Robinson, T., S. Ward-Close, and J. Potter, *A Comparison of beat-to-beat blood pressure variability in acute and subacute stroke patients with cerebral infarction.* Cerebrovasc Dis, 1997. **7**(2): p. 214-219.
43. Geeganage, C., M. Tracy, T. England, G. Sare, T. Moulin, F. Woimant*, et al.*, *Relationship between baseline blood pressure parameters (including mean pressure, pulse pressure, and variability) and early outcome after stroke: data from the Tinzaparin in Acute Ischaemic Stroke Trial (TAIST).* Stroke, 2011. **42**(2): p. 491-3.
44. Manning, L.S., A.K. Mistri, J. Potter, P.M. Rothwell, and T.G. Robinson, *Short-term blood pressure variability in acute stroke: post hoc analysis of the controlling hypertension and hypotension immediately post stroke and continue or stop post-stroke antihypertensives collaborative study trials.* Stroke, 2015. **46**(6): p. 1518-24.
45. Manning, L.S., P.M. Rothwell, J.F. Potter, and T.G. Robinson, *Prognostic Significance of Short-Term Blood Pressure Variability in Acute Stroke: Systematic Review.* Stroke, 2015. **46**(9): p. 2482-90.
46. Nagai, M. and K. Kario, *Visit-to-visit blood pressure variability, silent cerebral injury, and risk of stroke.* Am J Hypertens, 2013. **26**(12): p. 1369-76.
47. Bohm, M., H. Schumacher, D. Leong, G. Mancia, T. Unger, R. Schmieder*, et al.*, *Systolic blood pressure variation and mean heart rate is associated with cognitive dysfunction in patients with high cardiovascular risk.* Hypertension, 2015. **65**(3): p. 651-61.
48. McDonald, C., M.S. Pearce, S.R. Kerr, and J.L. Newton, *Blood pressure variability and cognitive decline in older people: a 5-year longitudinal study.* J Hypertens, 2017. **35**(1): p. 140-147.
49. Robinson, T.G., S.L. Dawson, P.J. Eames, R.B. Panerai, and J.F. Potter, *Cardiac baroreceptor sensitivity predicts long-term outcome after acute ischemic stroke.* Stroke, 2003. **34**(3): p. 705-12.

For peer review only

**Figure 1:** Study flow diagram.

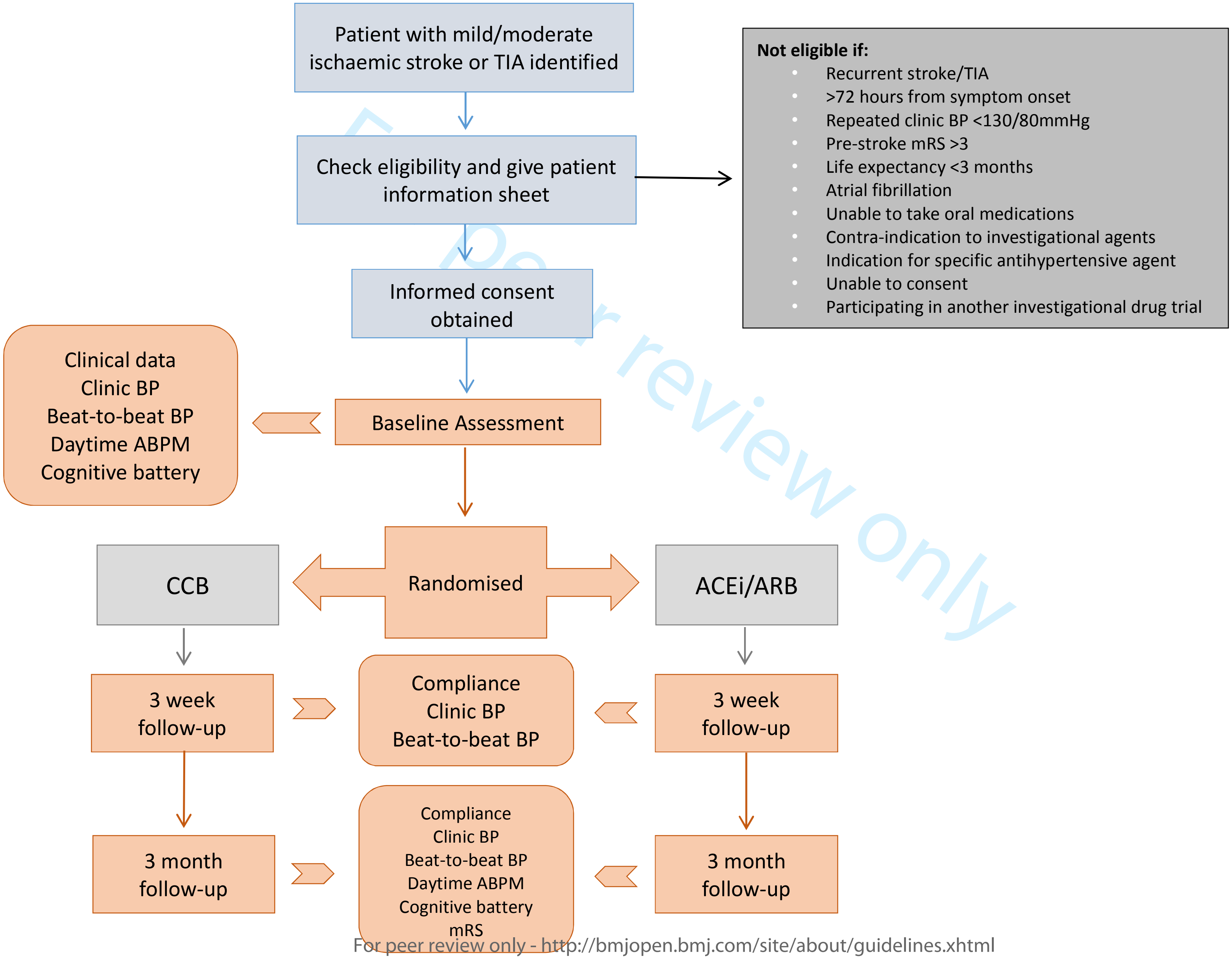


For peer review only - http://bmjopen.bmj.com/site/about/guidelines.xhtml

SPIRIT

STANDARD PROTOCOL ITEMS: RECOMMENDATIONS FOR INTERVENTIONAL TRIALS

SPIRIT 2013 Checklist: Recommended items to address in a clinical trial protocol and related documents*

| Section/item | Item No | Description | Addressed on page number |
|---|---|---|---|
| **Administrative information** | | | |
| Title | 1 | Descriptive title identifying the study design, population, interventions, and, if applicable, trial acronym | _______1_______ |
| Trial registration | 2a | Trial identifier and registry name. If not yet registered, name of intended registry | _______3_______ |
| | 2b | All items from the World Health Organization Trial Registration Data Set | _______________ |
| Protocol version | 3 | Date and version identifier | _______2_______ |
| Funding | 4 | Sources and types of financial, material, and other support | _______14______ |
| Roles and responsibilities | 5a | Names, affiliations, and roles of protocol contributors | _______1_______ |
| | 5b | Name and contact information for the trial sponsor | _______14_______ |
| | 5c | Role of study sponsor and funders, if any, in study design; collection, management, analysis, and interpretation of data; writing of the report; and the decision to submit the report for publication, including whether they will have ultimate authority over any of these activities | ______14______ |
| | 5d | Composition, roles, and responsibilities of the coordinating centre, steering committee, endpoint adjudication committee, data management team, and other individuals or groups overseeing the trial, if applicable (see Item 21a for data monitoring committee) | ______14_______ |



For peer review only - http://bmjopen.bmj.com/site/about/guidelines.xhtml

For peer review only

**Introduction**

| | | | |
|---|---|---|---|
| Background and rationale | 6a | Description of research question and justification for undertaking the trial, including summary of relevant studies (published and unpublished) examining benefits and harms for each intervention | ___4-6______ |
| | 6b | Explanation for choice of comparators | ____4-6_______ |
| Objectives | 7 | Specific objectives or hypotheses | ____6-7_______ |
| Trial design | 8 | Description of trial design including type of trial (eg, parallel group, crossover, factorial, single group), allocation ratio, and framework (eg, superiority, equivalence, noninferiority, exploratory) | ____7-8_______ |

**Methods: Participants, interventions, and outcomes**

| | | | |
|---|---|---|---|
| Study setting | 9 | Description of study settings (eg, community clinic, academic hospital) and list of countries where data will be collected. Reference to where list of study sites can be obtained | ____7-8_______ |
| Eligibility criteria | 10 | Inclusion and exclusion criteria for participants. If applicable, eligibility criteria for study centres and individuals who will perform the interventions (eg, surgeons, psychotherapists) | ____8______ |
| Interventions | 11a | Interventions for each group with sufficient detail to allow replication, including how and when they will be administered | ____10-12______ |
| | 11b | Criteria for discontinuing or modifying allocated interventions for a given trial participant (eg, drug dose change in response to harms, participant request, or improving/worsening disease) | ____10-11______ |
| | 11c | Strategies to improve adherence to intervention protocols, and any procedures for monitoring adherence (eg, drug tablet return, laboratory tests) | ____10-11______ |
| | 11d | Relevant concomitant care and interventions that are permitted or prohibited during the trial | ____10-11______ |
| Outcomes | 12 | Primary, secondary, and other outcomes, including the specific measurement variable (eg, systolic blood pressure), analysis metric (eg, change from baseline, final value, time to event), method of aggregation (eg, median, proportion), and time point for each outcome. Explanation of the clinical relevance of chosen efficacy and harm outcomes is strongly recommended | ____12_____ |
| Participant timeline | 13 | Time schedule of enrolment, interventions (including any run-ins and washouts), assessments, and visits for participants. A schematic diagram is highly recommended (see Figure) | ___10-12______ |



For peer review only - http://bmjopen.bmj.com/site/about/guidelines.xhtml

| | | | |
|---|---|---|---|
| Sample size | 14 | Estimated number of participants needed to achieve study objectives and how it was determined, including clinical and statistical assumptions supporting any sample size calculations | ____12_____ |
| Recruitment | 15 | Strategies for achieving adequate participant enrolment to reach target sample size | _____9________ |

**Methods: Assignment of interventions (for controlled trials)**

| | | | |
|---|---|---|---|
| Allocation: | | | |
| Sequence generation | 16a | Method of generating the allocation sequence (eg, computer-generated random numbers), and list of any factors for stratification. To reduce predictability of a random sequence, details of any planned restriction (eg, blocking) should be provided in a separate document that is unavailable to those who enrol participants or assign interventions | ______9______ |
| Allocation concealment mechanism | 16b | Mechanism of implementing the allocation sequence (eg, central telephone; sequentially numbered, opaque, sealed envelopes), describing any steps to conceal the sequence until interventions are assigned | ______9______ |
| Implementation | 16c | Who will generate the allocation sequence, who will enrol participants, and who will assign participants to interventions | ______9______ |
| Blinding (masking) | 17a | Who will be blinded after assignment to interventions (eg, trial participants, care providers, outcome assessors, data analysts), and how | _____N/A____ |
| | 17b | If blinded, circumstances under which unblinding is permissible, and procedure for revealing a participant's allocated intervention during the trial | _____N/A_____ |

**Methods: Data collection, management, and analysis**

| | | | |
|---|---|---|---|
| Data collection methods | 18a | Plans for assessment and collection of outcome, baseline, and other trial data, including any related processes to promote data quality (eg, duplicate measurements, training of assessors) and a description of study instruments (eg, questionnaires, laboratory tests) along with their reliability and validity, if known. Reference to where data collection forms can be found, if not in the protocol | ______10-12____ |
| | 18b | Plans to promote participant retention and complete follow-up, including list of any outcome data to be collected for participants who discontinue or deviate from intervention protocols | _____N/A_____ |



For peer review only - http://bmjopen.bmj.com/site/about/guidelines.xhtml

| | | | |
|---|---|---|---|
| Data management | 19 | Plans for data entry, coding, security, and storage, including any related processes to promote data quality (eg, double data entry; range checks for data values). Reference to where details of data management procedures can be found, if not in the protocol | ______14______ |
| Statistical methods | 20a | Statistical methods for analysing primary and secondary outcomes. Reference to where other details of the statistical analysis plan can be found, if not in the protocol | ______13-14____ |
| | 20b | Methods for any additional analyses (eg, subgroup and adjusted analyses) | ______13-14____ |
| | 20c | Definition of analysis population relating to protocol non-adherence (eg, as randomised analysis), and any statistical methods to handle missing data (eg, multiple imputation) | ______13-14____ |
| **Methods: Monitoring** | | | |
| Data monitoring | 21a | Composition of data monitoring committee (DMC); summary of its role and reporting structure; statement of whether it is independent from the sponsor and competing interests; and reference to where further details about its charter can be found, if not in the protocol. Alternatively, an explanation of why a DMC is not needed | ______14______ |
| | 21b | Description of any interim analyses and stopping guidelines, including who will have access to these interim results and make the final decision to terminate the trial | ______14______ |
| Harms | 22 | Plans for collecting, assessing, reporting, and managing solicited and spontaneously reported adverse events and other unintended effects of trial interventions or trial conduct | ______14____ |
| Auditing | 23 | Frequency and procedures for auditing trial conduct, if any, and whether the process will be independent from investigators and the sponsor | ______14______ |
| **Ethics and dissemination** | | | |
| Research ethics approval | 24 | Plans for seeking research ethics committee/institutional review board (REC/IRB) approval | ______14______ |
| Protocol amendments | 25 | Plans for communicating important protocol modifications (eg, changes to eligibility criteria, outcomes, analyses) to relevant parties (eg, investigators, REC/IRBs, trial participants, trial registries, journals, regulators) | ______14______ |



For peer review only - http://bmjopen.bmj.com/site/about/guidelines.xhtml

| | | | |
|---|---|---|---|
| Consent or assent | 26a | Who will obtain informed consent or assent from potential trial participants or authorised surrogates, and how (see Item 32) | _____9_____ |
| | 26b | Additional consent provisions for collection and use of participant data and biological specimens in ancillary studies, if applicable | _____N/A_____ |
| Confidentiality | 27 | How personal information about potential and enrolled participants will be collected, shared, and maintained in order to protect confidentiality before, during, and after the trial | _____14______ |
| Declaration of interests | 28 | Financial and other competing interests for principal investigators for the overall trial and each study site | _____14______ |
| Access to data | 29 | Statement of who will have access to the final trial dataset, and disclosure of contractual agreements that limit such access for investigators | ______14_____ |
| Ancillary and post-trial care | 30 | Provisions, if any, for ancillary and post-trial care, and for compensation to those who suffer harm from trial participation | ______N/A____ |
| Dissemination policy | 31a | Plans for investigators and sponsor to communicate trial results to participants, healthcare professionals, the public, and other relevant groups (eg, via publication, reporting in results databases, or other data sharing arrangements), including any publication restrictions | _____14______ |
| | 31b | Authorship eligibility guidelines and any intended use of professional writers | _____14______ |
| | 31c | Plans, if any, for granting public access to the full protocol, participant-level dataset, and statistical code | _____N/A_____ |
| **Appendices** | | | |
| Informed consent materials | 32 | Model consent form and other related documentation given to participants and authorised surrogates | _____N/A______ |
| Biological specimens | 33 | Plans for collection, laboratory evaluation, and storage of biological specimens for genetic or molecular analysis in the current trial and for future use in ancillary studies, if applicable | _____N/A_____ |

*It is strongly recommended that this checklist be read in conjunction with the SPIRIT 2013 Explanation & Elaboration for important clarification on the items. Amendments to the protocol should be tracked and dated. The SPIRIT checklist is copyrighted by the SPIRIT Group under the Creative Commons "Attribution-NonCommercial-NoDerivs 3.0 Unported" license.



For peer review only - http://bmjopen.bmj.com/site/about/guidelines.xhtml

# A Randomised Controlled Trial of a Calcium Channel or Angiotensin Converting Enzyme Inhibitor/Angiotensin Receptor Blocker Regime to Reduce Blood Pressure Variability following Ischaemic Stroke (CAARBS) – a protocol for a feasibility study


| | |
|---|---|
| Journal: | *BMJ Open* |
| Manuscript ID | bmjopen-2018-025301.R1 |
| Article Type: | Protocol |
| Date Submitted by the Author: | 25-Sep-2018 |
| Complete List of Authors: | Robinson, Tom; University of Leicester College of Medicine Biological Sciences and Psychology, Cardiovascular Sciences<br>Davison, William; University of East Anglia Faculty of Medicine and Health Sciences,<br>Rothwell, Peter; Stroke Prevention Research Unit, Nuffield Department of Clinical Neurosciences<br>Potter, John; University of East Anglia, Norwich Medical School; Norfolk and Norwich University Hospital, Stroke Research Group |
| <b>Primary Subject Heading</b>: | Cardiovascular medicine |
| Secondary Subject Heading: | Neurology, Geriatric medicine |
| Keywords: | blood pressure, blood pressure variability, Stroke < NEUROLOGY, cerebrovascular disease |
| | |


SCHOLARONE™
Manuscripts

# A Randomised Controlled Trial of a Calcium Channel or Angiotensin Converting Enzyme Inhibitor/Angiotensin Receptor Blocker Regime to Reduce Blood Pressure Variability following Ischaemic Stroke (CAARBS) – a protocol for a feasibility study

**Correspondence to:**

Dr William Davison

Clinical Research Fellow in Stroke Medicine

Bob Champion Research and Education Building,

James Watson Rd,

Norwich Research Park,

University of East Anglia,

Norwich,

NR4 7UQ

Tel: +44 (0) 1603 591245

Mail to: w.davison@uea.ac.uk

Keywords: blood pressure, blood pressure variability, stroke, cerebrovascular disease

Word count (excluding title page, refs, tables, figures): 3831 words

Authors:

Professor Thompson G Robinson, Professor of Stroke Medicine, University of Leicester, Leicester, UK

Dr William J Davison, Clinical Research Fellow in Stroke Medicine, University of East Anglia, Norwich, UK

Professor Peter M Rothwell, Action Research Professor of Clinical Neurology, University of Oxford, Oxford, UK

Professor John F Potter, Professor of Ageing & Stroke Medicine, University of East Anglia, Norwich, UK

Author Contributions:

For peer review only

For peer review only - http://bmjopen.bmj.com/site/about/guidelines.xhtml

T Robinson, J Potter and P Rothwell obtained funding for the project and designed the study as part of a programme of work on blood pressure variability and stroke. The protocol was written by T Robinson and W Davison and reviewed by the other contributing authors. This manuscript has been prepared by W Davison and adapted from protocol version 2.0 dated 20/7/18. The final manuscript has been reviewed by all authors and approved for submission/publication.

For peer review only

ABSTRACT

Introduction: Raised blood pressure (BP) is common post-stroke and is associated with a poor prognosis, yet trials of BP lowering in the immediate post-stroke period have not demonstrated a benefit. One possible explanation for this may be that BP variability (BPV) rather than absolute levels predicts outcome, as BPV is increased post-stroke and is associated with poor outcomes. Furthermore, there is evidence of distinct antihypertensive class effects on BPV despite similar BP lowering effects. However, whether BPV in the immediate post-stroke period is a therapeutic target has not been prospectively investigated.

The objectives of this trial are to assess the feasibility and safety of recruiting patients following an acute ischaemic stroke or transient ischaemic attack (TIA) to an interventional randomised controlled trial comparing the effects of two different antihypertensive drug classes on BPV. Secondary exploratory objectives are to assess if different therapeutic strategies have diverse effects on levels of BPV and if this has an impact on outcomes.

Methods: 150 adult patients with first-ever ischaemic stroke or TIA who require antihypertensive therapy for secondary prevention will be recruited within 72 hours of the event from stroke services across three sites. After baseline assessments they will be randomly assigned to treatment with a calcium channel blocker or angiotensin converting enzyme inhibitor/angiotensin receptor blocker based regimen and followed-up for a period of three months.

Ethics and dissemination: Ethical and regulatory approvals have been granted. Dissemination is planned via publication in peer-reviewed medical journals and presentation at relevant conferences.

Registration details: International standard randomised controlled trial number (ISRCTN) 10853487.

For peer review only

**Strengths and limitations of this study:**

- To our knowledge this is the first prospective randomised trial designed to assess the treatment of BPV following acute ischaemic stroke/TIA.
- The protocol incorporates multiple BP measurement methods.
- The chosen therapeutic interventions are in line with standard clinical practice for secondary stroke prevention.

- The trial is open-label which could bias the analysis of treatment effects on BPV and any impact on stroke outcomes, but these are secondary exploratory outcomes in this feasibility trial.

INTRODUCTION

**Background**

Raised BP is common after acute stroke with at least 75% of patients having a systolic BP (SBP) >130mmHg at hospital admission [1, 2]; SBP <130mmHg being the guideline target for secondary prevention following stroke [3]. Increased post-stroke BP is associated with poor prognosis [4, 5] and may result from raised intracranial pressure [6], increased sympathetic nervous system activity [7], abnormal baroreceptor sensitivity (BRS) [8], haematoma expansion [9], cerebral oedema [10], and a white-coat response [11]. A spontaneous BP decrease usually occurs 4 to 10 days post-ictus [12], but substantial BP reductions can be associated with cerebral hypoperfusion as a consequence of post-stroke dysautoregulation [13]. We have previously reported that both increased 24-hour and beat-to-beat BP levels following acute stroke are associated with a poor prognosis [14-16]. Subsequently, data from the International Stroke Trial has suggested a U-shaped relation between baseline SBP (within 48 hours of stroke) and short- (14-day mortality) and long-term (6-month death and dependency) outcomes; the lowest risk of death and dependency being at SBP of 150mmHg [17]. However, there is conflicting evidence regarding acute stroke hypertension treatment. Data from randomised controlled trials (RCTs) suggest that BP can be safely reduced after the acute stroke period, however, there seems to be no indication that doing so is beneficial. [18-23]. Indeed, the SCAST trial reported that it may actually be harmful, with a non-significant increased risk of poor 6-month functional outcome [23]. Therefore, Cochrane meta-analysis and guidelines state that optimal BP management in the context of initial stroke management remains uncertain [3, 24-26].

An alternative explanation for the lack of evidence that lowering elevated BP levels in acute stroke is beneficial may relate to the additional effects of BPV [27]. Current hypertension guidelines predominantly focus on mean, usually casual, BP measurements, dismissing BPV as random and merely an obstacle to the reliable estimation of usual BP. However, on ambulatory or home BP monitoring, which are recommended for the diagnosis and management of hypertension [28], mean BP is found to vary substantially [29], with the extent of this variation associated with visit-to-visit variability in clinic BP [30]. Indeed, there are many examples to support the potential importance of

For peer review only

BPV for vascular risk [30]. Firstly, the predictive value of estimated usual SBP and stroke risk falls with age [31], yet stroke incidence rises with age and the relative benefit of antihypertensive therapy is maintained in the elderly [32]. Secondly, an increased early-morning surge in BP is predictive of stroke, but is poorly associated with mean BP [33]. Thirdly, other causes of transient hypertension are recognised triggers of vascular events, including sympathetic overactivity and orthostatic hypertension [34]. Fourthly, in the majority of studies, there is no threshold of baseline SBP below which vascular risk stops falling (though evidence for BP below 115/75mmHg is very limited) [31], with antihypertensive therapy reducing risk even at 'normal' baseline SBP [35]. Fifthly, 'white-coat' hypertension, a common example of situational BPV, is associated with long-term target organ damage independent of mean BP [36]. Sixthly, though hypertension is a recognised risk factor for vascular dementia, there is limited evidence of reduced dementia risk in trials of antihypertensive therapy. However, a trial of calcium channel blockers (CCB), which have the most consistent effect on reducing BPV [37, 38], has shown a substantial reduction in the incidence of dementia [39]. Furthermore, in patients with Alzheimer's dementia BPV is increased compared to matched controls, with increased BPV being independently predictive of progressive cognitive decline in this patient group [40]. Finally, specific group differences in stroke risk are not accounted for by mean BP alone, for example in black individuals [41].

**Rationale for the study**

In a retrospective analysis of RCTs in a TIA population, visit-to-visit intra-individual BPV was a risk factor for stroke independent of the mean 'absolute' BP level, and perhaps of greater significance [30]. Additionally, within-visit systolic BPV, based on casual BP measurements, was correlated with visit-to-visit systolic BPV, but was a weak predictor of future vascular events [30]. Importantly, in a separate analysis it was demonstrated that BPV is reproducible and independent of confounding factors [42]. Increased BPV may also be an important predictor of short-term outcome following acute stroke. Robinson and colleagues have shown that beat-to-beat systolic BPV was greater in acute stroke compared to controls [43], and that high mean arterial and diastolic beat-to-beat BPV was associated with a worse prognosis [15]. Furthermore, in a post hoc analysis of the Tinzaparin in Acute Ischaemic Stroke Trial (TAIST), high systolic BPV from 3-6 casual BP readings, taken within 48 hours of symptom onset, was associated with an increase in death or early neurological deterioration at day 10 [44]. Conversely, a retrospective analysis of nearly 1,000 patients in the Continue or Stop Post-Stroke Antihypertensives Collaborative Study (COSSACS) and Controlling Hypertension and Hypotension Immediately Post Stroke (CHHIPS) trial did not demonstrate a significant association between systolic BPV based on two sets of three casual BP readings within 48

For peer review only

hours of stroke onset and two-week death and dependency [45]. Overall, a recent meta-analysis reported that increased systolic BPV, measured early from stroke onset, was associated with poor long-term functional outcome [46]. Furthermore, increased BPV may also relate to post-stroke cognitive outcomes with evidence suggesting an association with signs of cerebrovascular small vessel disease on neuroimaging [47], and deterioration in cognitive test scores [48, 49].

Clearly there is further scope to explore the relationship between BPV and outcome following acute stroke, in particular whether it has implications for therapeutic management in the immediate post-stroke period. Rothwell's group have explored the differential effects of BP-lowering therapies on BPV in a hypertensive population [37, 38]. Though clinical benefits with reduction in risk of stroke and coronary events were seen for all classes of antihypertensive agent, class-specific effects existed; CCBs reduce stroke risk to a greater extent than expected from mean SBP reduction alone, and beta-blockers (BB) to a smaller extent. A detailed analysis of the Anglo-Scandinavian Cardiac Outcomes Trial – Blood Pressure Lowering Arm (ASCOT-BPLA), comparing an amlodipine versus atenolol-based regime, and the Medical Research Council (MRC) trial, comparing an atenolol versus diuretic-based regime, reported opposite effects of CCB and BB on systolic BPV. In addition, this differential effect accounted for the disparity in observed effects on stroke risk and observed effects on mean SBP [38]. This was confirmed in a systematic review and meta-analysis of 389 RCTs which also demonstrated that BPV is reduced by non-loop diuretic drugs, but increased by angiotensin converting enzyme inhibitors (ACEI) and angiotensin receptor blockers (ARB) [37]. Again, the effects on systolic BPV were correlated with effects on stroke risk independent of differences in mean SBP [37]. Prospective trials to investigate these apparent medication class effects on BPV would be valuable, especially comparing CCB and ACEI/ARB which are typically the first-line antihypertensive drug classes. If, as anticipated, CCBs reduce BPV whereas ACEI and ARBs increase it this could be relevant after acute stroke, where normal cardiovascular autonomic and cerebrovascular autoregulatory pathways are impaired. BRS is important in the short-term regulation of the cardiovascular system, including BP, and is known to be impaired following acute ischaemic stroke [8], and associated with poor short and long-term prognosis [50]. In addition, it is well established that cerebral autoregulation (CA) is impaired, particularly following moderate to severe stroke [13]. As a consequence, cerebral perfusion is pressure-dependent, and therefore hypertensive episodes related to increased BPV may contribute to reperfusion injuries, for example post-ischaemic oedema and/or intracerebral haemorrhage. Conversely, hypotensive episodes associated with increased BPV in the presence of impaired CA may lead to secondary ischaemia, particularly in the absence of a good collateral circulation.

For peer review only

In conclusion, increased BPV is associated with a greater vascular risk, independent of mean BP and may predict poor outcomes after stroke. Furthermore, commonly used antihypertensive agents have different class effects on BPV which may in part explain the overall differential effects on stroke risk for similar absolute reductions in mean BP in a hypertensive population. Trials to investigate the potential therapeutic targeting of BPV and any potential benefit of doing so in acute stroke would be useful to address gaps in the current knowledge base.

**Study objectives**

The primary objective of this study is to determine feasibility of recruiting patients with acute stroke and TIA into an interventional randomised trial comparing the effect of different antihypertensive medication regimens on BPV.

Secondary feasibility objectives are:

- to determine the viability of measuring changes in BPV from baseline to 21 (±7) days and 90 (±14) days by treatment arm;
- to assess compliance rates with BPV measurement methods;
- to assess compliance rates with the investigational treatments;
- to identify serious adverse events (SAEs) associated with the interventions, including recurrent stroke/TIA, other cardiovascular events, death, and hospital readmission up to three months.

In addition to the feasibility objectives, exploratory outcomes that may be used in a future definitive RCT will be investigated. The proposed primary exploratory outcome will be modified Rankin score (mRS) at day 90.

Exploratory secondary outcomes are:

- mRS at day 21;
- National Institutes of Health Stroke Scale (NIHSS) at day 21;
- mean BP at day 21 and day 90;
- BPV at day 21 day and 90;
- Montreal cognitive assessment (MoCA) score at day 90.

METHODS AND ANALYSIS

**Study overview**

This study is a randomised, multi-centre, open-label parallel group study to determine the feasibility of conducting such a trial in an NHS setting to investigate class effects of antihypertensive medications on BPV in patients with acute ischaemic stroke or TIA. The aim is to evaluate barriers to recruitment, identify potential safety issues, and demonstrate that it is possible to detect differences in BPV over the proposed study duration. We also hope to investigate the potential therapeutic benefit of targeting BPV after acute ischaemic stroke/TIA in terms of functional outcome in order to help estimate the necessary sample size for a future definitive trial. A summary of the study design is provided in **Figure 1.** Recruitment commenced in January 2018 and is ongoing. The trial was prospectively registered: International standard randomised controlled trial number (ISRCTN) 10853487.

**Patient and Public Involvement**

The trial was conceived and designed without the involvement of patients or members of the public.

**Trial Participants**

All adult patients with clinically definite first-ever ischaemic stroke or TIA within 72 hours of onset will be considered for the trial.

**Inclusion Criteria**

- Age >18 years;
- First-ever clinically definite TIA and ischaemic stroke patients (NIHSS <10);
- Within 7 days of symptom onset (this criteria was initially within 72 hours of symptom onset, but was altered with a substantial amendment to the protocol to try and improve recruitment);
- Casual BP >130/80mmHg on repeat measurements;
- Ability to comply with randomly assigned BP-lowering regime and BP measurements;
- Able to understand written and verbal English;
- Able to give informed consent;
- Willing to allow his or her General Practitioner and consultant, if appropriate, to be notified of participation in the study.

For peer review only - http://bmjopen.bmj.com/site/about/guidelines.xhtml

**Exclusion Criteria**

The participant may not enter the trial if any of the following apply:

- Known definite contra-indication to BP-lowering regime or therapeutic agents;
- Swallowing difficulties which would preclude the taking of oral medication;
- Definite indication for BB, CCB, ACEI or ARB therapy;
- Significant pre-stroke dependency (mRS >3);
- Co-existing life-threatening condition with life expectancy <3 months;
- Previous participation in this trial or current participation in another investigational drug trial;
- Atrial fibrillation;
- Female participants who are pregnant, lactating or planning pregnancy during the course of the study;
- Unable to understand written and verbal English;
- Cannot give informed consent.

**Identification of participants**

First-ever TIA and minor ischaemic stroke patients referred to and assessed by the in- and/ or out-patients stroke services at three centres within 7 days of symptom onset will be identified by the treating clinician and/ or the research team. If the patient provides verbal consent to be considered for the study then their medical records will then be assessed against the study inclusion and exclusion criteria. Patients known to be hypertensive and on treatment prior to their cerebrovascular event should have their antihypertensive medications suspended at admission, in keeping with standard practice at the recruiting centres, unless there is a specific indication for them to continue. Where treatment is suspended and the patient is willing to be considered for the trial then they are potentially eligible for inclusion provided other inclusion/exclusion criteria are not violated. Once a potential participant has been confirmed to be eligible then research staff will approach the individual to discuss the study in more detail, provide a Participant Information Sheet, and seek written informed consent.

**Obtaining informed consent**

The participant must personally sign and date the latest approved version of the informed consent form, countersigned by a delegated member of the research team, before any study specific procedures are performed. Written and verbal versions of the participant information sheet and

For peer review only

Informed consent form will be presented to the participants detailing no less than: the exact nature of the study; the implications and constraints of the protocol; the known side effects and any risks involved in taking part. It will be clearly stated that the participant is free to withdraw from the study at any time for any reason without prejudice to future care, and with no obligation to give the reason for withdrawal. The person who obtained the consent must be suitably qualified and experienced, and have been authorised to do so by the Chief/Principal Investigator as detailed on the Delegation of Authority and Signature log for the study. The original signed form will be retained at the study site within the Investigator Site File. A copy of the signed Informed Consent will be given to participants and a copy retained in their medical notes.

**Randomisation**

After the baseline assessments eligible patients will be randomised using a computer generated protocol, in blocks of four, to a dihydropyridine CCB or ACEI/ARB-based regime. The study treatment will be dispensed at the baseline visit, but treatment will not be commenced within 48 hours of the qualifying event in keeping with current recommended practice. The actual therapeutic agent used will be at the discretion of the treating clinician, but dictated by the class of therapy that the participant is assigned to. Prescription of the medication will be done by the treating clinician and the initial supply will be dispensed by the treating hospital or community pharmacy in accordance with the hospital's policy for providing discharge or out-patient medication. Further supplies will be provided by the participant's GP. Unblinding will not be necessary as there is an open-label study design.

**Interventions to be measured**

***Routine clinical data***

The following routine clinical information and investigation results will be obtained from the medical notes and by participant interview:

- Demographics (including age, sex, ethnicity, height and weight, smoking and alcohol habits);
- Past medical history and family history of cardiovascular disease;
- Concomitant medications;
- NIHSS;
- mRS (including pre-morbid mRS);
- Oxford Community Stroke Project and TOAST classification;
- Laboratory tests (including full blood count, clotting, urea, electrolytes, creatinine, estimated glomerular filtration rate, total cholesterol, and random glucose);

For peer review only

- 12-lead ECG (± 24-hour ECG if performed);
- Imaging investigations (including neuroimaging (CT or MRI), carotid ultrasound, and cardiac echocardiography where applicable).

***Blood pressure measurements***

Baseline casual BP will be calculated as a mean of two sets of three supine brachial BP readings taken 10 minutes apart, using a UA767 BP monitor (referred to as enhanced casual BP).

Three consecutive periods of 10-min beat-to-beat non-invasive BP monitoring in the supine position using the middle finger of the non-hemiparetic hand will be recorded with a Finometer device. The servo adjust mechanism of the Finometer will be switched off during the recording period, but applied at 10 minute intervals during the monitoring period.

Daytime ABPM will be performed using a SpaceLabs 90207 monitor, programmed to measure BP at 20-minute intervals. Daytime is defined as between 0700-2200 hours.

***Cognitive testing***

A battery of cognitive tests will be performed. This will include the MoCA screening test which is established for use after cerebrovascular events, augmented with the Albert's line test for inattention, the Motor Neuron Disease Behavioural Instrument (MiND-B) for frontal cognitive symptoms, and the Geriatric Depression Scale (GDS) to exclude significant concurrent anxiety/depression.

**Follow-up Assessments**

These will be undertaken at day 21 (±7 days) and day 90 (±14 days) in the trial centre or where the patient is resident at the time (including the hospital ward, rehabilitation facility, or their own home). Interventions that will repeated at these follow-up visits are summarised in **Table 1**.

Additional follow-up interventions to assess the trial feasibility and safety will include assessment of treatment compliance using a self-reported questionnaire and tablet count (with compliance defined as ≥80%), and assessment of any side-effects and SAEs. Patients randomised to the ACEI/ARB arm will have repeat renal function blood tests at the first follow-up visit in line with standard practice to ensure their safety. In those patients failing to reach casual supine/sitting BP target of <130/ 80mmHg, the medical assessor at the follow-up visit will advise about altering BP-lowering treatment and this will be communicated to the participant's GP. The first-line change will be to increase the study regime medication (i.e. CCB or ACEI/ARB) to twice the starting dose. If the patient is on the maximum dose of the study regime medication already, then the second-line change will be to add a thiazide-like diuretic. If a third-line change is required then Spironolactone or an alpha-blocker will be added to the combination of study medication and thiazide-like diuretic.

For peer review only

After the second follow-up visit ongoing management of the patient's BP will be taken over by the GP.

**Table 1: Summary of trial procedures**

| Procedures | Visits | | | |
|---|---|---|---|---|
| | **Screening** | **Baseline** | **21 (±7) days** | **90 (±14) days** |
| Informed consent | | **X** | | |
| Demographics | | **X** | | |
| Medical history | | **X** | | |
| Concomitant medications | | **X** | **X** | **X** |
| ECG | | **X** | | |
| Clinical investigation results (bloods tests, CT/MRI scan results) | | **X** | | |
| Eligibility assessment | **X** | | | |
| Randomisation | | **X** | | |
| Dispensing of study drugs | | **X** | | |
| Treatment compliance | | | **X** | **X** |
| Blood test for renal function in ACEI/ARB group | | | **X** | |
| NIHSS | | **X** | **X** | **X** |
| mRS | | **X**[1] | **X** | **X** |
| MoCA | | **X** | | **X** |
| Albert's line test | | **X** | | **X** |
| MiND-B | | **X** | | **X** |
| GDS | | **X** | | **X** |
| Enhanced casual BP | | **X** | **X** | **X** |
| Beat-to-beat BP measurements | | **X** | **X** | **X** |
| Daytime ABPM | | **X** | | **X** |
| SAEs | | | **X** | **X**[2] |

[1] Including Premorbid mRS

[2] SAEs at Day 90 followed-up until resolution

**Outcome measurements**

For peer review only

1. Primary feasibility outcome measure:

   Recruitment and retention rates at three months from the screening and management logs, and reasons for ineligibility or non-inclusion of those screened but not recruited.

2. Secondary feasibility outcome measures:

   (a) changes in BPV from baseline to 21 (+7) days and 90 (+14) days by treatment arm;

   (b) proportions of participants achieving ≥80% treatment compliance by treatment arm;

   (c) treatment discontinuation rates;

   (d) completion and failure rates of BPV measurements at 21 (+7) days and 90 (+14) days;

   (e) serious adverse event rates by treatment arm.

3. Exploratory outcome measures:

   (a) mRS at 90 (+14) days by treatment arm;

   (b) mRS at 21 (+7) days by treatment arm;

   (c) NIHSS at 21 (+7) days by treatment arm;

   (d) differences in mean BP at 21 (+7) days and 90 (+14) days by treatment arm;

   (e) differences in BPV at 21 (+7) days and 90 (+14) days by treatment arm;

   (f) differences in MoCA score at 90 (+14) days by treatment arm.

**Sample size calculation**

A feasibility study of 150 patients (64 patients per group with a 15% drop-out rate) will have an 80% power at the 5% significance level of detecting an 8mmHg difference in systolic BPV between the CCB and ACEI/ARB-based regimes, assuming a mean systolic BPV SD of 14.97mmHg in the CCB arm and 16.95mmHg in the ACEI/ARB arm [37].

**Data analysis plan**

The primary objective is assessment of feasibility. This will focus on recruitment and retention rates, compliance, change in BPV, and safety of the intervention. Exploratory analysis of the effect of the proposed intervention on BPV and stroke outcome will be done as a secondary objective.

***Recruitment and Retention***

The total numbers of patients screened, the proportion recruited, and the proportion completing follow-up will be determined. Reasons for ineligibility, non-inclusion, and withdrawal will be analysed using descriptive statistical methods.

***Assessment of the intervention***

For peer review only

Compliance with the intervention will be assessed by the proportion of participants who achieve ≥80% adherence to the trial medication and the proportion of participants who have all BP measurements recorded successfully.

The feasibility of detecting changes in BPV will also be assessed. Within-individual systolic, diastolic and mean BPV will be expressed as the standard deviation, coefficient of variation, average real variability, and variation independent of the mean calculated from all BP measurements: enhanced casual, beat-to-beat measurements (each 10 minute recording and the total 30 minute recording), and daytime ABPM [42]. Changes in within-individual BPV from baseline to the follow-up time points will be analysed using a general linear model. The size of the mean difference will be estimated for each approach and compared to select the most appropriate measure for a future study.

***Safety***

Rates of serious adverse events, including recurrent stroke/TIA, other cardiovascular events, death, and hospitalization will be recorded up to 3 months. A descriptive comparison will be undertaken to compare the rates, but no formal hypothesis testing will be undertaken.

***Exploratory Analyses***

Mean BP will be calculated from enhanced casual measurements. Change in mean BP from baseline to follow-up by treatment arm will be compared using an independent samples T test.

An assessment of treatment effect on BPV will be undertaken stratified according to treatment arm. A general linear model will be used with BPV as the dependent variable and treatment arm as the independent variable, adjusting for baseline BP and diagnosis (stroke vs. TIA). Each expression of BPV as described above will be analysed.

Exploratory assessment of treatment effect on stroke outcome will be undertaken by comparing between-group differences in mRS and MoCA score at follow-up using independent samples T tests or a non-parametric test if the assumptions of the t-test are violated.

## ETHICS AND DISSEMINATION

This study was granted ethical approval in England (London - Central Research Ethics Committee, REC 17/LO/1427) and clinical trial authorisation from the Medicines and Healthcare products Regulatory Agency (EudraCT number 2017-002560-41). Subsequently the trial was approved by the Health Research Authority. Study oversight will be conducted through regular meetings of a Trial

For peer review only

Steering Committee and a separate Safety Committee, both of which will include independent representatives. If it is felt that the risk to participants is significant or unacceptable the Safety Committee can recommend to early termination of the trial.

The proposed investigational medicinal products are antihypertensives that are already in routine use and so their safety profiles are known. The medications are expected to lower the BP of participants. Therefore, in line with accepted stroke guidelines we will only recruit patients with uncontrolled BP (>130/ 80mmHg) who would otherwise require antihypertensive treatment for secondary stroke prevention. Medications that inhibit the renin-angiotensin system are known to potentially cause kidney dysfunction in patients with unrecognised renal artery stenosis. To ensure the safety of patients commenced on these medications a blood test for kidney function will be done at the 2 to 4-week follow-up which is in keeping with standard practice.

The trial will be conducted in accordance with the Declaration of Helsinki and the ICH Guidelines for Good Clinical Practice. All participants will provide written informed consent. Data will be collected and handled in line with sponsor standard operating procedures and NHS Trust policies. Electronic data will be anonymised and all data will be kept under secure conditions. Professor Robinson will act as data custodian.

Dissemination of the study results is planned via publication in peer-reviewed medical journals and presentation at relevant scientific conferences. Any reporting will adhere to the CONSORT statement extension for pilot and feasibility trials. We do not intend to employ professional writers.

Competing Interests: Professor Robinson and Professor Rothwell are both NIHR Senior Investigators.

Funding: This work was supported by a programme grant awarded jointly by The Stroke Association and The British Heart Foundation (Ref: TSA BHF 2012/01).

The study is sponsored by the University of Leicester. The sponsor has not had a role in the design or implementation of the study, nor the writing of this protocol.

Contact details for the sponsor:

Dr Michelle Muessel

Research Governance Manager

For peer review only

Research Governance Office

Research and Enterprise Division

University of Leicester

Leicester General Hospital

Gwendolen Rd

Leicester LE5 4PW

Tel: 0116 2584099/2584867

Mail to: uolsponsor@le.ac.uk

For peer review only

**References**

1. IST Collaborative Group, *The International Stroke Trial (IST): a randomised trial of aspirin, subcutaneous heparin, both, or neither among 19435 patients with acute ischaemic stroke. .* Lancet, 1997. **349**(9065): p. 1569-81.
2. CAST (Chinese Acute Stroke Trial) Collaborative Group, *CAST: randomised placebo-controlled trial of early aspirin use in 20,000 patients with acute ischaemic stroke.* Lancet, 1997. **349**(9066): p. 1641-9.
3. Intercollegiate Stroke Working Party. *National Clinical Guideline for Stroke 5th ed.* 2016 [cited 2017 17th July 2017]; Available from: https://www.rcplondon.ac.uk/guidelines-policy/stroke-guidelines.
4. Willmot, M., J. Leonardi-Bee, and P.M. Bath, *High blood pressure in acute stroke and subsequent outcome: a systematic review.* Hypertension, 2004. **43**(1): p. 18-24.
5. Tikhonoff, V., H. Zhang, T. Richart, and J.A. Staessen, *Blood pressure as a prognostic factor after acute stroke.* Lancet Neurol, 2009. **8**(10): p. 938-48.
6. Fodstad, H., P.J. Kelly, and M. Buchfelder, *History of the cushing reflex.* Neurosurgery, 2006. **59**(5): p. 1132-7; discussion 1137.
7. Chamorro, A., S. Amaro, M. Vargas, V. Obach, A. Cervera, M. Gomez-Choco*, et al.*, *Catecholamines, infection, and death in acute ischemic stroke.* J Neurol Sci, 2007. **252**(1): p. 29-35.
8. Robinson, T.G., M. James, J. Youde, R. Panerai, and J. Potter, *Cardiac baroreceptor sensitivity is impaired after acute stroke.* Stroke, 1997. **28**(9): p. 1671-6.
9. Ohwaki, K., E. Yano, H. Nagashima, M. Hirata, T. Nakagomi, and A. Tamura, *Blood pressure management in acute intracerebral hemorrhage: relationship between elevated blood pressure and hematoma enlargement.* Stroke, 2004. **35**(6): p. 1364-7.
10. Qureshi, A.I., *Acute hypertensive response in patients with stroke: pathophysiology and management.* Circulation, 2008. **118**(2): p. 176-87.
11. Carlberg, B., K. Asplund, and E. Hagg, *High blood pressure in acute stroke--is it white coat hypertension?* J Intern Med, 1990. **228**(3): p. 291-2.
12. Britton, M., A. Carlsson, and U. de Faire, *Blood pressure course in patients with acute stroke and matched controls.* Stroke, 1986. **17**(5): p. 861-4.
13. Aries, M.J., J.W. Elting, J. De Keyser, B.P. Kremer, and P.C. Vroomen, *Cerebral autoregulation in stroke: a review of transcranial Doppler studies.* Stroke, 2010. **41**(11): p. 2697-704.
14. Robinson, T., A. Waddington, S. Ward-Close, N. Taub, and J. Potter, *The predictive role of 24-hour compared to casual blood pressure levels on outcome following acute stroke.* Cerebrovasc Dis, 1997. **7**: p. 264-272.

15. Dawson, S.L., B.N. Manktelow, T.G. Robinson, R.B. Panerai, and J.F. Potter, *Which parameters of beat-to-beat blood pressure and variability best predict early outcome after acute ischemic stroke?* Stroke, 2000. **31**(2): p. 463-468.
16. Webb, A.J.S., S. Mazzucco, L. Li, and P.M. Rothwell, *Prognostic Significance of Blood Pressure Variability on Beat-to-Beat Monitoring After Transient Ischemic Attack and Stroke.* Stroke, 2018. **49**(1): p. 62-67.
17. Leonardi-Bee, J., P.M. Bath, S.J. Phillips, P.A. Sandercock, and I.S.T.C. Group, *Blood pressure and clinical outcomes in the International Stroke Trial.* Stroke, 2002. **33**(5): p. 1315-20.
18. Schrader, J., S. Luders, A. Kulschewski, J. Berger, W. Zidek, J. Treib*, et al.*, *The ACCESS Study: evaluation of Acute Candesartan Cilexetil Therapy in Stroke Survivors.* Stroke, 2003. **34**(7): p. 1699-703.
19. Anderson, C.S., Y. Huang, J.G. Wang, H. Arima, B. Neal, B. Peng*, et al.*, *Intensive blood pressure reduction in acute cerebral haemorrhage trial (INTERACT): a randomised pilot trial.* Lancet Neurol, 2008. **7**(5): p. 391-9.
20. Potter, J.F., T.G. Robinson, G.A. Ford, A. Mistri, M. James, J. Chernova*, et al.*, *Controlling hypertension and hypotension immediately post-stroke (CHHIPS): a randomised, placebo-controlled, double-blind pilot trial.* Lancet Neurol, 2009. **8**(1): p. 48-56.
21. Bath, P.M., R.H. Martin, Y. Palesch, D. Cotton, S. Yusuf, R. Sacco*, et al.*, *Effect of telmisartan on functional outcome, recurrence, and blood pressure in patients with acute mild ischemic stroke: a PRoFESS subgroup analysis.* Stroke, 2009. **40**(11): p. 3541-6.
22. Robinson, T.G., J.F. Potter, G.A. Ford, C.J. Bulpitt, J. Chernova, C. Jagger*, et al.*, *Effects of antihypertensive treatment after acute stroke in the Continue or Stop Post-Stroke Antihypertensives Collaborative Study (COSSACS): a prospective, randomised, open, blinded-endpoint trial.* Lancet Neurol, 2010. **9**(8): p. 767-75.
23. Sandset, E.C., P.M. Bath, G. Boysen, D. Jatuzis, J. Korv, S. Luders*, et al.*, *The angiotensin-receptor blocker candesartan for treatment of acute stroke (SCAST): a randomised, placebo-controlled, double-blind trial.* Lancet, 2011. **377**(9767): p. 741-50.
24. Bath, P.M. and K. Krishnan, *Interventions for deliberately altering blood pressure in acute stroke.* Cochrane Database Syst Rev, 2014(10): p. CD000039.
25. National Institute for Health and Care Excellence. *Stroke and transient ischaemic attack in over 16s: diagnosis and initial management*. 2008 [cited 2017 17th July 2017]; Available from: https://www.nice.org.uk/guidance/CG68.
26. Kernan, W.N., B. Ovbiagele, H.R. Black, D.M. Bravata, M.I. Chimowitz, M.D. Ezekowitz*, et al.*, *Guidelines for the prevention of stroke in patients with stroke and transient ischemic attack: a guideline for healthcare professionals from the American Heart Association/American Stroke Association.* Stroke, 2014. **45**(7): p. 2160-236.
27. Rothwell, P.M., *Limitations of the usual blood-pressure hypothesis and importance of variability, instability, and episodic hypertension.* Lancet, 2010. **375**(9718): p. 938-48.
28. National Institute for Health and Care Excellence. *Hypertension in adults: diagnosis and management*. 2011 [cited 2017 17th July 2017]; Available from: www.nice.org.uk/guidance/cg127.
29. Wizner, B., D.G. Dechering, L. Thijs, N. Atkins, R. Fagard, E. O'Brien*, et al.*, *Short-term and long-term repeatability of the morning blood pressure in older patients with isolated systolic hypertension.* J Hypertens, 2008. **26**(7): p. 1328-35.
30. Rothwell, P.M., S.C. Howard, E. Dolan, E. O'Brien, J.E. Dobson, B. Dahlof*, et al.*, *Prognostic significance of visit-to-visit variability, maximum systolic blood pressure, and episodic hypertension.* Lancet, 2010. **375**(9718): p. 895-905.
31. Lewington, S., R. Clarke, N. Qizilbash, R. Peto, R. Collins, and C. Prospective Studies, *Age-specific relevance of usual blood pressure to vascular mortality: a meta-analysis of individual data for one million adults in 61 prospective studies.* Lancet, 2002. **360**(9349): p. 1903-13.

For peer review only

32. Beckett, N.S., R. Peters, A.E. Fletcher, J.A. Staessen, L. Liu, D. Dumitrascu, *et al.*, *Treatment of hypertension in patients 80 years of age or older.* N Engl J Med, 2008. **358**(18): p. 1887-98.
33. Kario, K., K. Shimada, and T.G. Pickering, *Clinical implication of morning blood pressure surge in hypertension.* J Cardiovasc Pharmacol, 2003. **42 Suppl 1**: p. S87-91.
34. Kario, K., *Orthostatic hypertension: a measure of blood pressure variation for predicting cardiovascular risk.* Circ J, 2009. **73**(6): p. 1002-7.
35. Progress Collaborative Group, *Randomised trial of a perindopril-based blood-pressure-lowering regimen among 6,105 individuals with previous stroke or transient ischaemic attack.* Lancet, 2001. **358**(9287): p. 1033-41.
36. Mancia, G., M. Bombelli, R. Facchetti, F. Madotto, F. Quarti-Trevano, H. Polo Friz, *et al.*, *Long-term risk of sustained hypertension in white-coat or masked hypertension.* Hypertension, 2009. **54**(2): p. 226-32.
37. Webb, A.J., U. Fischer, Z. Mehta, and P.M. Rothwell, *Effects of antihypertensive-drug class on interindividual variation in blood pressure and risk of stroke: a systematic review and meta-analysis.* Lancet, 2010. **375**(9718): p. 906-15.
38. Rothwell, P.M., S.C. Howard, E. Dolan, E. O'Brien, J.E. Dobson, B. Dahlof, *et al.*, *Effects of beta blockers and calcium-channel blockers on within-individual variability in blood pressure and risk of stroke.* Lancet Neurol, 2010. **9**(5): p. 469-80.
39. Forette, F., M.L. Seux, J.A. Staessen, L. Thijs, W.H. Birkenhager, M.R. Babarskiene, *et al.*, *Prevention of dementia in randomised double-blind placebo-controlled Systolic Hypertension in Europe (Syst-Eur) trial.* Lancet, 1998. **352**(9137): p. 1347-51.
40. Lattanzi, S., F. Brigo, F. Vernieri, and M. Silvestrini, *Visit-to-visit variability in blood pressure and Alzheimer's disease.* J Clin Hypertens (Greenwich), 2018. **20**(5): p. 918-924.
41. Duprez, D.A., D.R. Jacobs, Jr., P.L. Lutsey, D. Herrington, D. Prime, P. Ouyang, *et al.*, *Race/ethnic and sex differences in large and small artery elasticity--results of the multi-ethnic study of atherosclerosis (MESA).* Ethn Dis, 2009. **19**(3): p. 243-50.
42. Howard, S.C. and P.M. Rothwell, *Reproducibility of measures of visit-to-visit variability in blood pressure after transient ischaemic attack or minor stroke.* Cerebrovasc Dis, 2009. **28**(4): p. 331-40.
43. Robinson, T., S. Ward-Close, and J. Potter, *A Comparison of beat-to-beat blood pressure variability in acute and subacute stroke patients with cerebral infarction.* Cerebrovasc Dis, 1997. **7**(2): p. 214-219.
44. Geeganage, C., M. Tracy, T. England, G. Sare, T. Moulin, F. Woimant, *et al.*, *Relationship between baseline blood pressure parameters (including mean pressure, pulse pressure, and variability) and early outcome after stroke: data from the Tinzaparin in Acute Ischaemic Stroke Trial (TAIST).* Stroke, 2011. **42**(2): p. 491-3.
45. Manning, L.S., A.K. Mistri, J. Potter, P.M. Rothwell, and T.G. Robinson, *Short-term blood pressure variability in acute stroke: post hoc analysis of the controlling hypertension and hypotension immediately post stroke and continue or stop post-stroke antihypertensives collaborative study trials.* Stroke, 2015. **46**(6): p. 1518-24.
46. Manning, L.S., P.M. Rothwell, J.F. Potter, and T.G. Robinson, *Prognostic Significance of Short-Term Blood Pressure Variability in Acute Stroke: Systematic Review.* Stroke, 2015. **46**(9): p. 2482-90.
47. Nagai, M. and K. Kario, *Visit-to-visit blood pressure variability, silent cerebral injury, and risk of stroke.* Am J Hypertens, 2013. **26**(12): p. 1369-76.
48. Bohm, M., H. Schumacher, D. Leong, G. Mancia, T. Unger, R. Schmieder, *et al.*, *Systolic blood pressure variation and mean heart rate is associated with cognitive dysfunction in patients with high cardiovascular risk.* Hypertension, 2015. **65**(3): p. 651-61.
49. McDonald, C., M.S. Pearce, S.R. Kerr, and J.L. Newton, *Blood pressure variability and cognitive decline in older people: a 5-year longitudinal study.* J Hypertens, 2017. **35**(1): p. 140-147.


For peer review only

50. Robinson, T.G., S.L. Dawson, P.J. Eames, R.B. Panerai, and J.F. Potter, *Cardiac baroreceptor sensitivity predicts long-term outcome after acute ischemic stroke.* Stroke, 2003. **34**(3): p. 705-12.

**Figure Legends**

**Figure 1:** Study flow diagram.

For peer review only

**Figure 1:** Study flow diagram.

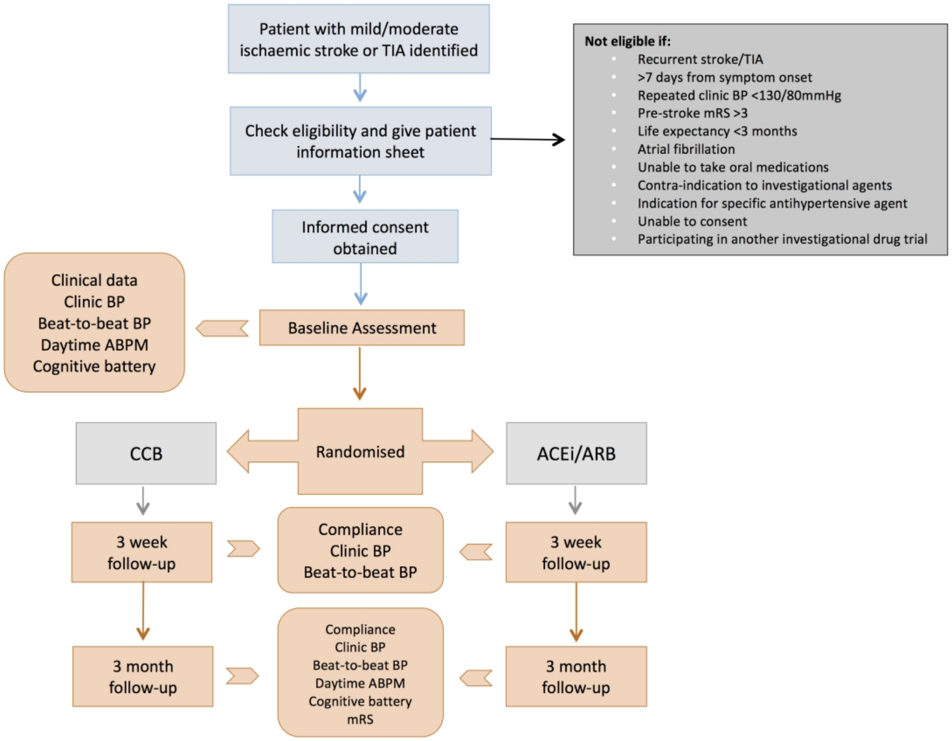


Figure 1: Study flow diagram

239x194mm (300 x 300 DPI)

SPIRIT

STANDARD PROTOCOL ITEMS: RECOMMENDATIONS FOR INTERVENTIONAL TRIALS

SPIRIT 2013 Checklist: Recommended items to address in a clinical trial protocol and related documents*

| Section/item | Item No | Description | Addressed on page number |
|---|---|---|---|
| **Administrative information** | | | |
| Title | 1 | Descriptive title identifying the study design, population, interventions, and, if applicable, trial acronym | 1 |
| Trial registration | 2a | Trial identifier and registry name. If not yet registered, name of intended registry | 3 |
| | 2b | All items from the World Health Organization Trial Registration Data Set | |
| Protocol version | 3 | Date and version identifier | 2 |
| Funding | 4 | Sources and types of financial, material, and other support | 14 |
| Roles and responsibilities | 5a | Names, affiliations, and roles of protocol contributors | 1 |
| | 5b | Name and contact information for the trial sponsor | 14 |
| | 5c | Role of study sponsor and funders, if any, in study design; collection, management, analysis, and interpretation of data; writing of the report; and the decision to submit the report for publication, including whether they will have ultimate authority over any of these activities | 14 |
| | 5d | Composition, roles, and responsibilities of the coordinating centre, steering committee, endpoint adjudication committee, data management team, and other individuals or groups overseeing the trial, if applicable (see Item 21a for data monitoring committee) | 14 |



For peer review only - http://bmjopen.bmj.com/site/about/guidelines.xhtml

For peer review only

**Introduction**

| | | | |
|---|---|---|---|
| Background and rationale | 6a | Description of research question and justification for undertaking the trial, including summary of relevant studies (published and unpublished) examining benefits and harms for each intervention | ___4-6______ |
| | 6b | Explanation for choice of comparators | ____4-6_______ |
| Objectives | 7 | Specific objectives or hypotheses | ____6-7_______ |
| Trial design | 8 | Description of trial design including type of trial (eg, parallel group, crossover, factorial, single group), allocation ratio, and framework (eg, superiority, equivalence, noninferiority, exploratory) | ____7-8_______ |

**Methods: Participants, interventions, and outcomes**

| | | | |
|---|---|---|---|
| Study setting | 9 | Description of study settings (eg, community clinic, academic hospital) and list of countries where data will be collected. Reference to where list of study sites can be obtained | ____7-8_______ |
| Eligibility criteria | 10 | Inclusion and exclusion criteria for participants. If applicable, eligibility criteria for study centres and individuals who will perform the interventions (eg, surgeons, psychotherapists) | ____8______ |
| Interventions | 11a | Interventions for each group with sufficient detail to allow replication, including how and when they will be administered | ____10-12______ |
| | 11b | Criteria for discontinuing or modifying allocated interventions for a given trial participant (eg, drug dose change in response to harms, participant request, or improving/worsening disease) | ____10-11______ |
| | 11c | Strategies to improve adherence to intervention protocols, and any procedures for monitoring adherence (eg, drug tablet return, laboratory tests) | ____10-11______ |
| | 11d | Relevant concomitant care and interventions that are permitted or prohibited during the trial | ____10-11______ |
| Outcomes | 12 | Primary, secondary, and other outcomes, including the specific measurement variable (eg, systolic blood pressure), analysis metric (eg, change from baseline, final value, time to event), method of aggregation (eg, median, proportion), and time point for each outcome. Explanation of the clinical relevance of chosen efficacy and harm outcomes is strongly recommended | ____12_____ |
| Participant timeline | 13 | Time schedule of enrolment, interventions (including any run-ins and washouts), assessments, and visits for participants. A schematic diagram is highly recommended (see Figure) | ___10-12______ |



For peer review only - http://bmjopen.bmj.com/site/about/guidelines.xhtml

| | | | |
|---|---|---|---|
| Sample size | 14 | Estimated number of participants needed to achieve study objectives and how it was determined, including clinical and statistical assumptions supporting any sample size calculations | ____12_____ |
| Recruitment | 15 | Strategies for achieving adequate participant enrolment to reach target sample size | _____9________ |

**Methods: Assignment of interventions (for controlled trials)**

| | | | |
|---|---|---|---|
| Allocation: | | | |
| Sequence generation | 16a | Method of generating the allocation sequence (eg, computer-generated random numbers), and list of any factors for stratification. To reduce predictability of a random sequence, details of any planned restriction (eg, blocking) should be provided in a separate document that is unavailable to those who enrol participants or assign interventions | ______9______ |
| Allocation concealment mechanism | 16b | Mechanism of implementing the allocation sequence (eg, central telephone; sequentially numbered, opaque, sealed envelopes), describing any steps to conceal the sequence until interventions are assigned | ______9______ |
| Implementation | 16c | Who will generate the allocation sequence, who will enrol participants, and who will assign participants to interventions | ______9______ |
| Blinding (masking) | 17a | Who will be blinded after assignment to interventions (eg, trial participants, care providers, outcome assessors, data analysts), and how | _____N/A____ |
| | 17b | If blinded, circumstances under which unblinding is permissible, and procedure for revealing a participant's allocated intervention during the trial | _____N/A_____ |

**Methods: Data collection, management, and analysis**

| | | | |
|---|---|---|---|
| Data collection methods | 18a | Plans for assessment and collection of outcome, baseline, and other trial data, including any related processes to promote data quality (eg, duplicate measurements, training of assessors) and a description of study instruments (eg, questionnaires, laboratory tests) along with their reliability and validity, if known. Reference to where data collection forms can be found, if not in the protocol | ______10-12____ |
| | 18b | Plans to promote participant retention and complete follow-up, including list of any outcome data to be collected for participants who discontinue or deviate from intervention protocols | _____N/A_____ |



For peer review only - http://bmjopen.bmj.com/site/about/guidelines.xhtml

| | | | |
|---|---|---|---|
| Data management | 19 | Plans for data entry, coding, security, and storage, including any related processes to promote data quality (eg, double data entry; range checks for data values). Reference to where details of data management procedures can be found, if not in the protocol | _____14______ |
| Statistical methods | 20a | Statistical methods for analysing primary and secondary outcomes. Reference to where other details of the statistical analysis plan can be found, if not in the protocol | _____13-14____ |
| | 20b | Methods for any additional analyses (eg, subgroup and adjusted analyses) | _____13-14____ |
| | 20c | Definition of analysis population relating to protocol non-adherence (eg, as randomised analysis), and any statistical methods to handle missing data (eg, multiple imputation) | _____13-14____ |
| **Methods: Monitoring** | | | |
| Data monitoring | 21a | Composition of data monitoring committee (DMC); summary of its role and reporting structure; statement of whether it is independent from the sponsor and competing interests; and reference to where further details about its charter can be found, if not in the protocol. Alternatively, an explanation of why a DMC is not needed | _____14______ |
| | 21b | Description of any interim analyses and stopping guidelines, including who will have access to these interim results and make the final decision to terminate the trial | _____14______ |
| Harms | 22 | Plans for collecting, assessing, reporting, and managing solicited and spontaneously reported adverse events and other unintended effects of trial interventions or trial conduct | _____14____ |
| Auditing | 23 | Frequency and procedures for auditing trial conduct, if any, and whether the process will be independent from investigators and the sponsor | _____14______ |
| **Ethics and dissemination** | | | |
| Research ethics approval | 24 | Plans for seeking research ethics committee/institutional review board (REC/IRB) approval | _____14______ |
| Protocol amendments | 25 | Plans for communicating important protocol modifications (eg, changes to eligibility criteria, outcomes, analyses) to relevant parties (eg, investigators, REC/IRBs, trial participants, trial registries, journals, regulators) | _____14______ |



For peer review only - http://bmjopen.bmj.com/site/about/guidelines.xhtml

| | | | |
|---|---|---|---|
| Consent or assent | 26a | Who will obtain informed consent or assent from potential trial participants or authorised surrogates, and how (see Item 32) | _____9_____ |
| | 26b | Additional consent provisions for collection and use of participant data and biological specimens in ancillary studies, if applicable | _____N/A_____ |
| Confidentiality | 27 | How personal information about potential and enrolled participants will be collected, shared, and maintained in order to protect confidentiality before, during, and after the trial | _____14______ |
| Declaration of interests | 28 | Financial and other competing interests for principal investigators for the overall trial and each study site | _____14______ |
| Access to data | 29 | Statement of who will have access to the final trial dataset, and disclosure of contractual agreements that limit such access for investigators | ______14_____ |
| Ancillary and post-trial care | 30 | Provisions, if any, for ancillary and post-trial care, and for compensation to those who suffer harm from trial participation | ______N/A____ |
| Dissemination policy | 31a | Plans for investigators and sponsor to communicate trial results to participants, healthcare professionals, the public, and other relevant groups (eg, via publication, reporting in results databases, or other data sharing arrangements), including any publication restrictions | _____14______ |
| | 31b | Authorship eligibility guidelines and any intended use of professional writers | _____14______ |
| | 31c | Plans, if any, for granting public access to the full protocol, participant-level dataset, and statistical code | _____N/A_____ |
| **Appendices** | | | |
| Informed consent materials | 32 | Model consent form and other related documentation given to participants and authorised surrogates | _____N/A______ |
| Biological specimens | 33 | Plans for collection, laboratory evaluation, and storage of biological specimens for genetic or molecular analysis in the current trial and for future use in ancillary studies, if applicable | _____N/A_____ |

*It is strongly recommended that this checklist be read in conjunction with the SPIRIT 2013 Explanation & Elaboration for important clarification on the items. Amendments to the protocol should be tracked and dated. The SPIRIT checklist is copyrighted by the SPIRIT Group under the Creative Commons "Attribution-NonCommercial-NoDerivs 3.0 Unported" license.



For peer review only - http://bmjopen.bmj.com/site/about/guidelines.xhtml
